# Defect‐Engineered BiO_2‐X_ Nanosheets Mediate Sono‐Thermomechanical ECM Remodeling for Hepatocellular Carcinoma Immunotherapy

**DOI:** 10.1002/advs.77337

**Published:** 2026-08-29

**Authors:** Huimin Tian, Shen Zhang, Bolin Wu, Yichi Chen, Haitao Shang, Haoyan Tan, Chunyue Wang, Weichen Xu, Huixiong Xu, Haohao Yin, Wen Cheng

**Affiliations:** ^1^ Department of Ultrasound Harbin Medical University Cancer Hospital Harbin Heilongjiang People's Republic of China; ^2^ Department of Ultrasound Institute of Ultrasound in Medicine and Engineering Zhongshan Hospital Fudan University Shanghai People's Republic of China; ^3^ Department of Medical Ultrasound Center of Minimally Invasive Treatment for Tumor Shanghai Tenth People's Hospital School of Medicine Tongji University Shanghai People's Republic of China

**Keywords:** extracellular matrix, fibronectin 1, hepatocellular carcinoma, immunotherapy, ultrasound

## Abstract

Immune checkpoint blockade for hepatocellular carcinoma (HCC) is frequently limited by the extracellular matrix (ECM). Through transcriptomic profiling, we identify that acquired anti‐PD‐1 resistance in HCC models is closely associated with prominent Fibronectin 1 (Fn1) upregulation within the tumor microenvironment. To address these interconnected physical and biological barriers, we developed an ultrasound‐responsive nanoplatform utilizing oxygen‐defect‐abundant 2D BiO_2‐X_ nanosheets loaded with Fn1‐targeted small interfering RNA (BiO_2‐X_/siFn1). Introducing oxygen vacancies into ultrathin BiO_2‐X_ alters its band structure, enhancing sono‐thermomechanical energy conversion under localized acoustic excitation. This synchronized sono‐thermomechanical effect physically remodels the dense collagen matrix, enhancing intratumoral permeation and spatially facilitating immune cell infiltration without relying on extreme hyperthermia. Concurrently, sonothermal‐sonomechanical synergistic enhancement of intracellular delivery of siFn1 efficiently downregulates Fn1 expression. This genetic intervention deprives detached tumor cells of integrin‐mediated focal adhesion survival signals, resensitizing them to anoikis and effectively suppressing ECM‐associated pulmonary metastasis. The BiO_2‐X_/siFn1 nanoplatform significantly increased the recruitment of CD8^+^ T cells in tumors and restored the therapeutic effect of inhibiting PD‐1 in HCC by combining macroscopic physical ECM remodeling with precise molecular blocking of mechanical conduction pathways. This defect‐engineered sonosensitization strategy modulates the solid tumor microenvironment and overcomes mechanically induced immunotherapy resistance.

## Graphical abstract

Herein, an ultrasound‐responsive BiO_2‐X_/siFn1 nanoplatform is developed to overcome anti‐PD‐1 resistance in hepatocellular carcinoma. Upon ultrasound irradiation, the platform downregulates fibronectin expression, while its sonothermal effect softens the dense extracellular matrix and activates antitumor immunity in the tumor microenvironment, thereby promoting T‐cell infiltration and restoring tumor sensitivity to anti‐PD‐1 therapy.

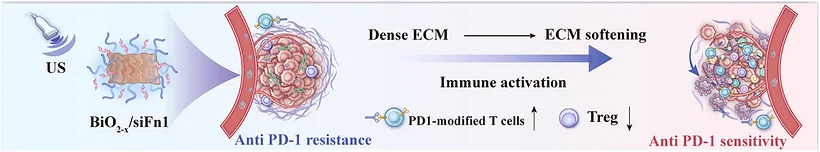

## 1 Introduction

Hepatocellular carcinoma (HCC) constitutes a leading factor responsible for cancer‐associated mortality across the world, frequently developing against a background of chronic inflammation and severe liver cirrhosis [1, 2, 3]. Although immune checkpoint blockade, particularly therapeutic agents directed against the PD‐1/PD‐L1 signaling axis, have significantly advanced the systemic management of HCC, only a limited proportion of patients, generally around 20%–30%, derive meaningful clinical benefit from these treatments [4, 5, 6]. This limitation is frequently hindered by primary or acquired resistance rooted in the profoundly fibrotic and immunosuppressive tumor microenvironment (TME) characteristic of HCC [7]. The pathological accumulation of extracellular matrix (ECM) components—including fibrillar collagens, hyaluronic acid, and heavily cross‐linked glycoproteins—constructs a formidable biophysical barricade [8].

This elevated interstitial fluid pressure and dense stromal architecture mechanically exclude the intratumoral infiltration of cytotoxic CD8^+^ T cells, effectively establishing an immune‐excluded or “cold” phenotype [9]. Furthermore, this desmoplastic niche provides potent mechanobiological survival cues to malignant cells [10]. Specifically, the excessive deposition of fibronectin (FN) engages integrin‐mediated mechanotransduction pathways (such as FAK/Src and PI3K/AKT signaling), effectively protecting detached tumor cells against anoikis, a programmed cell death process caused by the loss of adhesion between cells and the ECM, thereby facilitating metastatic spread [11, 12, 13]. Therefore, safely dismantling the ECM barrier and severing its associated survival signaling networks are critical prerequisites for reinvigorating anti‐PD‐1 efficacy.

In this study, transcriptome sequencing analysis was performed on a PD‐1‐resistant HCC model. It was found that Fibronectin 1 (Fn1) gene expression was markedly increased, and the identified differentially expressed genes (DEGs) were predominantly associated with pathways involved in ECM regulation. Conventional pharmacological approaches to degrade the ECM, such as systemic enzymatic digestions (e.g., collagenase or hyaluronidase) or chemical inhibitors targeting stromal cell activation, are frequently compromised by poor deep‐tissue penetration, rapid biological clearance, and non‐specific systemic toxicities [14]. In contrast, focused ultrasound (US) has emerged as a compelling, non‐invasive physical modality capable of penetrating deep anatomical tissues to provide high spatiotemporal control over local energy deposition [15]. However, efficiently transducing acoustic energy into precise, localized matrix disruption—primarily via acoustic cavitation, microstreaming, and shear stress—without relying on indiscriminate, extreme hyperthermia that risks damaging adjacent healthy hepatic parenchyma, requires the rational design of advanced sonosensitive nanomaterials.

To address this nanotechnological and engineering challenge, we rationally developed defect‐rich, ultrathin two‐dimensional (2D) bismuth oxide (BiO_2‐X_) nanosheets as an extraordinarily efficient US‐responsive nanoplatform. The deliberate introduction of abundant oxygen vacancies within the BiO_2‐X_ crystal lattice significantly alters its electronic band structure [16, 17]. These crystalline defects act as electron trap centers, narrowing the bandgap, suppressing electron‐hole recombination, and enhancing phonon scattering [17]. This defect engineering structurally optimizes the 2D material to efficiently harvest acoustic waves and amplify sono‐thermomechanical energy conversion at mild, clinically safe US intensities [18]. Furthermore, the 2D sheet‐like morphology provides a vast specific surface area for optimal acoustic impedance matching [19]. To enable targeted genetic intervention and ensure physiological stability during systemic circulation, these nanosheets were surface‐modified with a multifaceted polymer, methoxy poly(ethylene glycol)‐linked dextran‐grafted polyethylenimine (mPEG‐d‐PEI) [17, 20, 21]. The external PEGylation confers necessary stealth properties against macrophage clearance, while the internal cationic PEI segments effectively condense small interfering RNA targeting Fn1 (forming the BiO_2‐X_/siFn1 complex) via electrostatic interactions, protecting the fragile nucleic acid payload from premature nuclease degradation [22].

Upon systemic administration and targeted local acoustic excitation at the tumor site, the synchronized sono‐thermomechanical stress generated by the BiO_2‐X_ nanosheets induces controlled, localized cavitation. This physical mechanism directly and structurally remodels the dense collagenous ECM, relaxing the stroma and reducing interstitial fluid pressure to facilitate the deep intratumoral permeation of both nanotherapeutics and recruited effector T cells [23, 24, 25]. Concurrently, US‐mediated sonoporation transiently increases cellular membrane permeability, facilitating the efficient cytosolic delivery and rapid endosomal escape of siFn1 [23, 26, 27]. This cascade achieves robust, targeted downregulation of Fn1 expression at the messenger RNA (mRNA) level. The precise molecular blockade of ECM‐derived mechanotransduction deprives malignant cells of essential focal adhesion signals, resensitizing them to anoikis and substantially suppressing distant hepatic or pulmonary metastasis [28]. By coupling macroscopic physical stromal disruption with the targeted transcriptional silencing of mechanobiological survival pathways, the BiO_2‐X_/siFn1 nanoplatform fundamentally reprograms the immunosuppressive TME. This combined physical and genetic modulation synergizes directly with systemic anti‐PD‐1 therapy, transforming immunologically “cold” tumors into “hot” environments, thus promoting a sustained antitumor immune response and prolonging survival in refractory HCC models (Figure 1).

**FIGURE 1:**
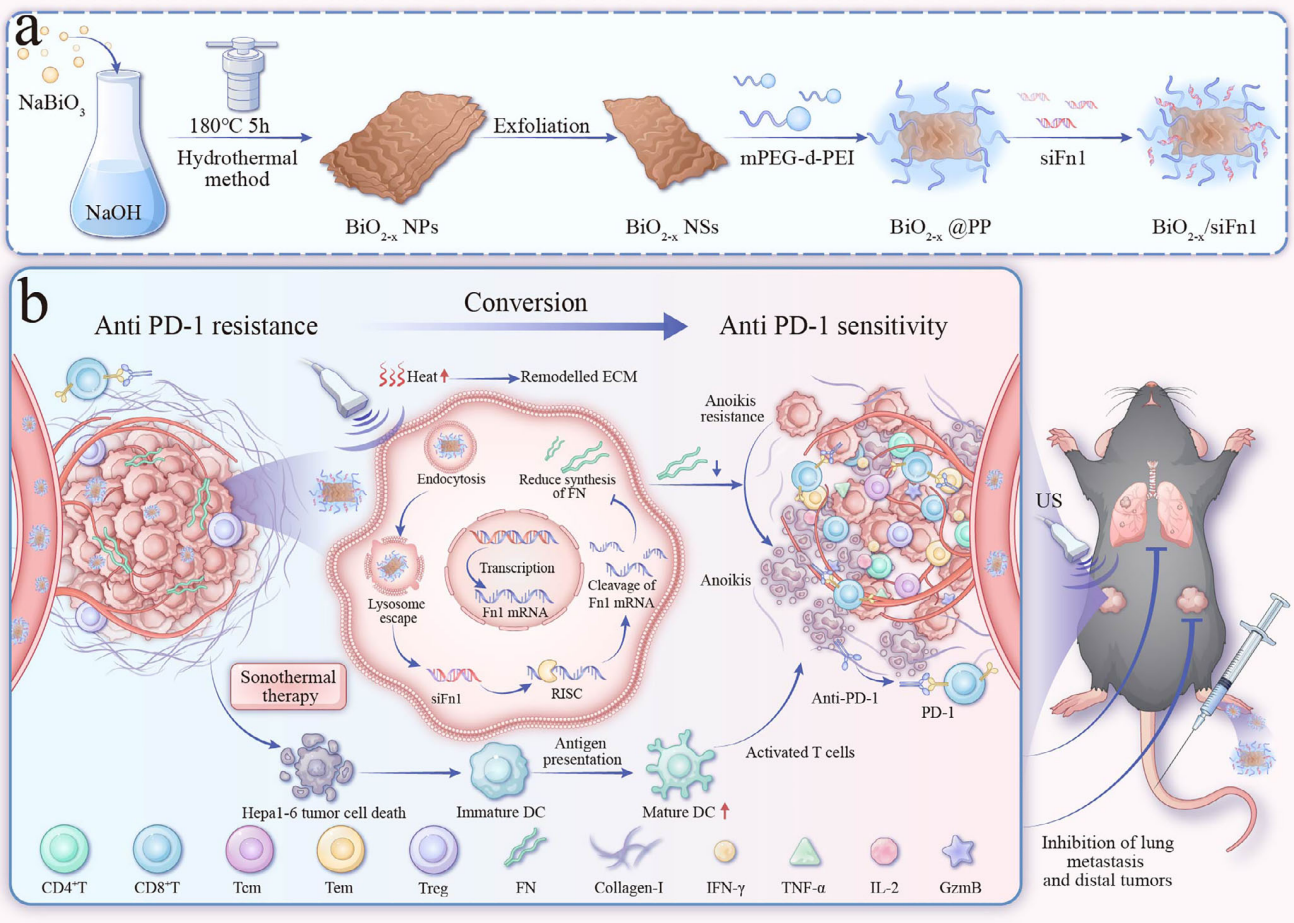
Design and mechanism of US‐responsive BiO_2‐X_/siFn1 for inhibiting HCC. The preparation scheme of BiO_2‐X_/siFn1 delivery system and the schematic diagram of BiO_2‐X_/siFn1 + US treatment is used to achieve immune infiltration and tumor killing after ECM remodeling in HCC.

## 2 Results

### 2.1 Transcriptomic Reveals Fn1‐Mediated ECM Remodeling Drives Acquired Resistance to PD‐1 Blockade Therapy

To characterize the changes in the TME accompanying resistance to PD‐1 antibody treatment, we established a mouse model of anti‐PD‐1 antibody‐resistant HCC and systematically revealed the dynamic remodeling of the TME following the development of resistance [29]. Although anti‐PD‐1 monoclonal antibody (mAb) treatment initially suppressed tumor growth, five tumors progressively lost responsiveness and developed acquired resistance within 30 days (Figure 2a,b). RNA sequencing was performed on anti‐PD‐1‐resistant tumor tissues collected on day 30, followed by comparative analysis against the control group. Specifically, volcano plot and heat map analyses identified a total of 6572 DEGs, comprising 2963 upregulated and 3609 downregulated genes (Figure 2c,d), indicating remarkable transcriptomic alterations in tumors following the acquisition of resistance to PD‐1 blockade therapy. In addition, the tumor samples were further analyzed using Gene Ontology (GO) and Kyoto Encyclopedia of Genes and Genomes (KEGG) pathway enrichment. The results showed that after PD‐1 antibody treatment resistance, DEGs were predominantly concentrated in pathways associated with the ECM (Figure 2e and Figure S1a). These results indicate that PD‐1 antibody treatment resistance is related to changes in ECM in the TME.

**FIGURE 2:**
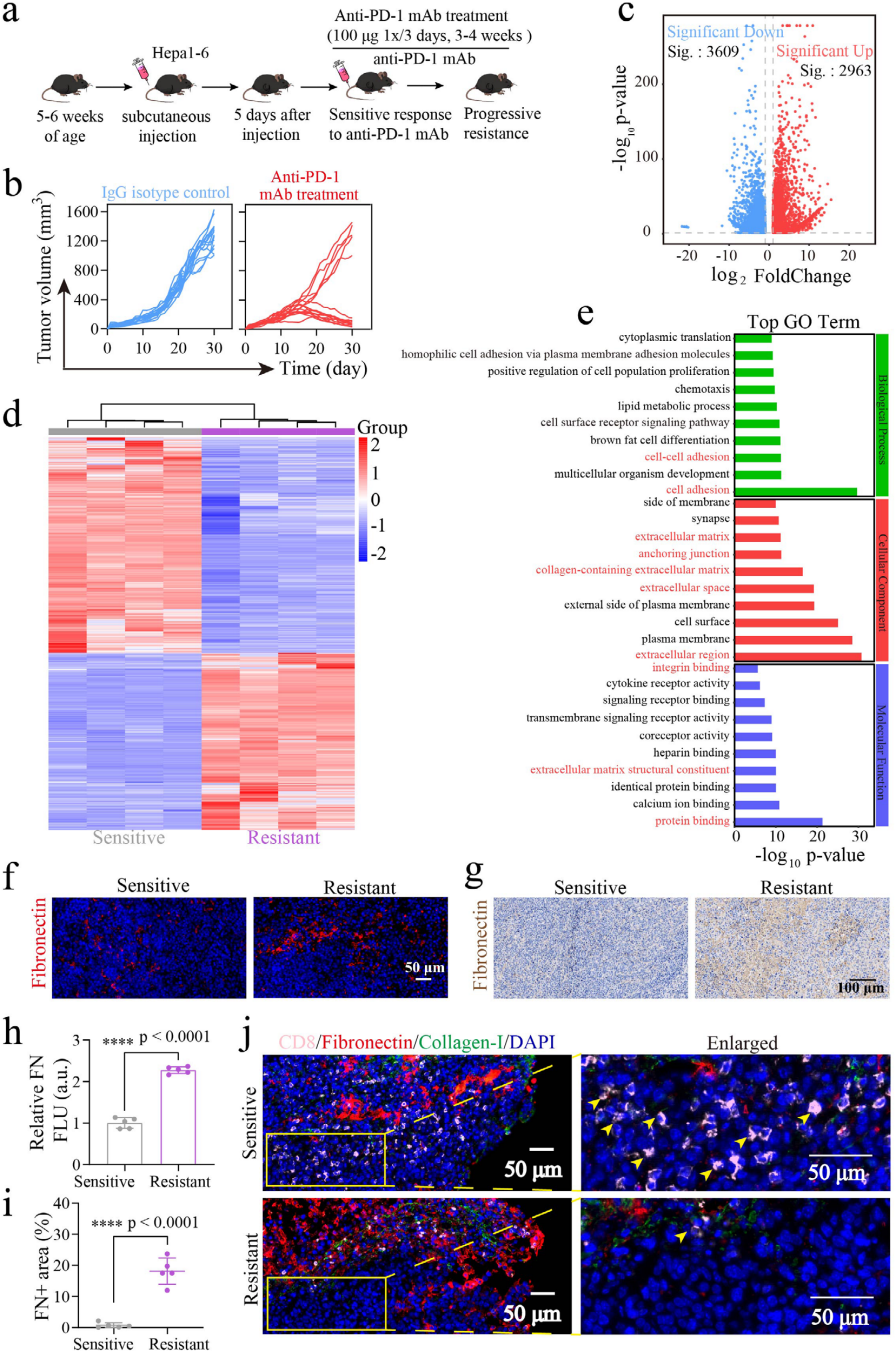
Analysis of factors leading to drug resistance in PD‐1 antibody therapy. (a, b) Schematic illustration of the anti‐PD‐1 treatment regimen and tumor growth curves in the mouse model (*n* = 15). (c) Volcano plots depicting gene expression alterations in resistant tumors compared with sensitive tumors (*n* = 4). (d) Clustering analysis of DEGs between the Resistant and Sensitive groups (*n* = 4). (e) Markedly enriched GO terms (top 30, *n* = 4). (f) Representative IF images of FN results in the tumor slices from the mice. (g) Representative IHC staining images of FN results in the tumor. (h) Quantification of relative fluorescence intensity of FN (*n* = 5). (i) The FN positive (FN+) area (%) calculated from Panel (*n* = 5). (j) Representative mIF micrographs of tumor tissues demonstrating CD8 (pink), FN (red), Collagen‐I (green) and DAPI (blue). Arrows: CD8^+^ cells. Statistical significance for (h, i) was determined using a two‐tailed unpaired Student's *t*‐test. Values are shown as means ± standard deviation (SD). ^*^ *p* < 0.05, ^**^ *p* < 0.01, ^***^ *p* < 0.001, ^****^ *p* < 0.0001, n.s., no significance.

In addition, sequencing results showed that the gene expression of Fn1, one of the main components of ECM, was up‐regulated. Immunofluorescence (IF) staining and immunohistochemistry (IHC) staining were performed to observe the increased expression of FN in tumor tissues resistant to PD‐1 antibody treatment, suggesting that the accumulation of ECM components may be a key feature of the drug‐resistant microenvironment (Figure 2f–i).

To investigate the causal relationship between FN protein and resistance to PD‐1 antibody therapy, we transfected Hepa1‐6 cells with short hairpin RNA (shRNA) targeting the Fn1 gene to establish the Hepa1‐6‐shFn1 cell line. Meanwhile, a cell line transfected with non‐targeting shRNA was constructed as the control group (designated Hepa1‐6‐shControl). We first verified the downregulated expression of FN protein in Hepa1‐6‐shFn1 cells (Figure S1b,c). Subsequently, we explored the functional role of FN protein in mediating tumor resistance to PD‐1 antibody therapy (Figure S1d). C57BL/6 mice received subcutaneous inguinal injections of tumor cells transduced with either shFn1 or shControl, followed by administration of PD‐1 antibody, and tumor growth dynamics were continuously monitored. The results demonstrated that tumor growth was markedly suppressed in the shFn1 group relative to the control group, and no tumors developed resistance to anti‐PD‐1 treatment in the shFn1 group (Figure S1e). Collectively, these findings indicate that upregulated FN protein expression acts as a driving factor underlying tumor resistance to PD‐1 antibody therapy.

Collagen‐I is the main component of ECM, so multiplex immunofluorescence (mIF) staining was performed on tumor tissues. The results showed that collagen‐I was localized around the tumor, and in the drug‐resistant group, CD8^+^ T cells accumulated in the peritumoral stroma. Conversely, in the group sensitive to anti‐PD‐1 therapy, abundant T cell infiltration was observed within the tumor (Figure 2j). In summary, the ECM in PD‐1 antibody‐resistant tumor tissues hinders T cell infiltration, and the effective removal of the matrix barrier while increasing the infiltration of activated T cells will effectively improve the resistance problem.

### 2.2 Fabrication and Characterization of BiO_2‐X_/siFn1

Motivated by the transcriptomic analysis identifying Fn1 as a critical therapeutic target, we engineered a defect‐rich, US‐responsive BiO_2‐X_/siFn1 nanoplatform to fundamentally remodel the resistant TME. BiO_2‐X_ nanoplates (BiO_2‐X_ NPs) were first prepared through a simple hydrothermal method. Subsequent ultrasonic fragmentation converted the nanoplates into ultrathin, vacancy‐rich BiO_2‐X_ nanosheets (BiO_2‐X_ NSs/BiO_2‐X_). The overall fabrication procedure and structural characteristics of BiO_2‐X_ are illustrated in Figure 3a,b.

**FIGURE 3:**
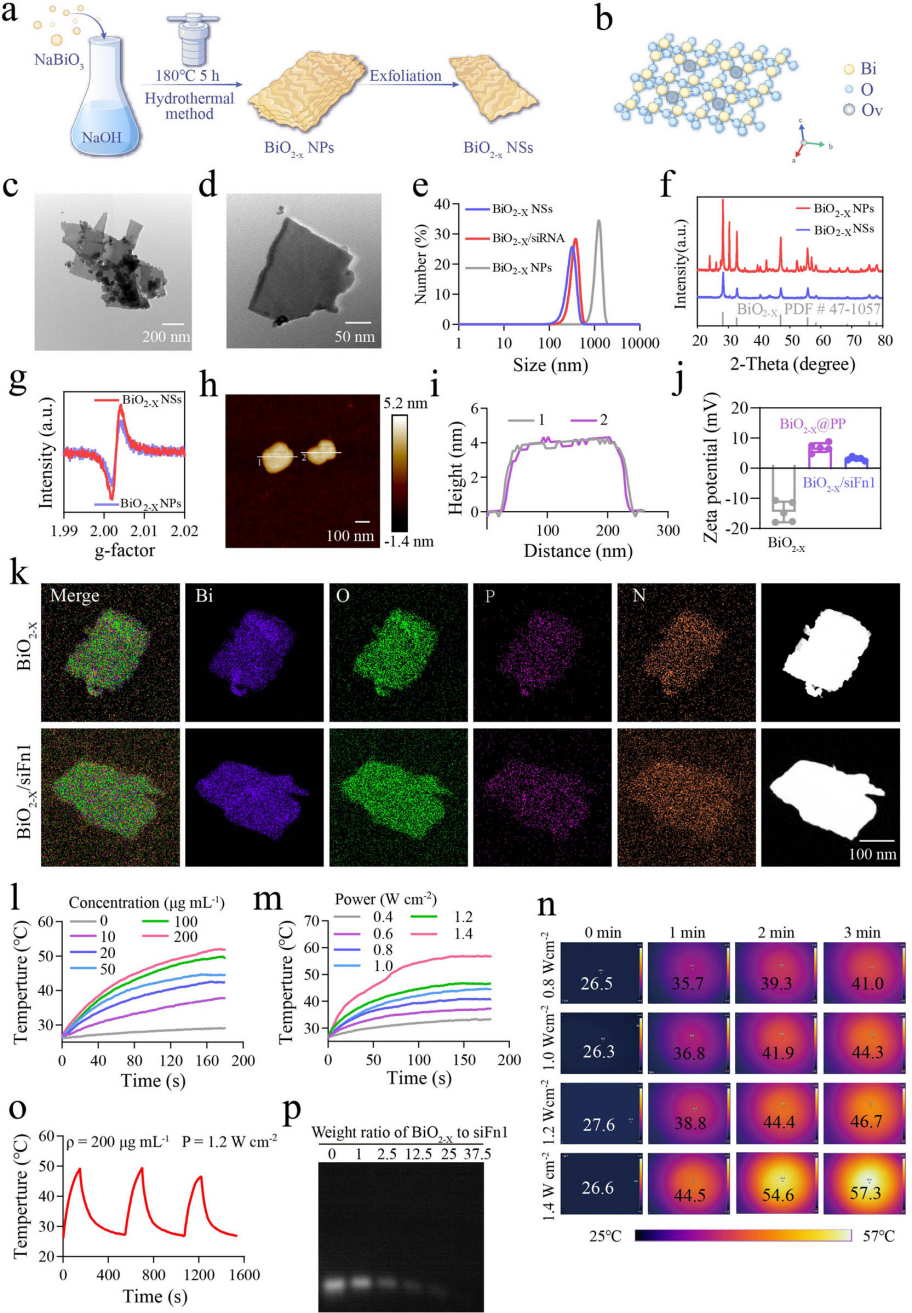
Preparation and characterization of BiO_2‐X_/siFn1. (a) Schematic illustration of synthetic process of BiO_2−X_ NSs. (b) Schematic diagram of structure of BiO_2−X_ NSs. (c) TEM image of BiO_2‐X_ NPs. (d) TEM image of BiO_2‐X_ NSs. (e) Hydrodynamic size distributions of BiO_2‐X_ NSs, BiO_2‐X_/siFn1, and BiO_2‐X_ NPs. (f) The X‐ray diffraction spectra of BiO_2‐X_ NSs and BiO_2‐X_ NPs. (g) EPR spectra of BiO_2‐X_ NSs and BiO_2‐X_ NPs. (h) AFM height images of the prepared BiO_2‐X_ NSs and (i) corresponding height profiles of BiO_2‐X_ NSs. (j) Zeta potential of BiO_2‐X_, BiO_2‐X_@PP, and BiO_2‐X_/siFn1 (*n* = 5). (k) The corresponding element mapping of the BiO_2‐X_ NSs and BiO_2‐X_/siFn1. (l) Concentration‐dependent sonothermal properties of BiO_2‐X_ in H_2_O with US irradiation. (m) Power‐dependent sonothermal properties of BiO_2‐X_ in H_2_O with BiO_2‐X_ concentration of 50 µg mL^−1^. (n) Corresponding thermal images of BiO_2‐X_ after irradiation with US. (o) Sonothermal cycling performance of the BiO_2‐X_ dispersion over three consecutive US on/off cycles at an intensity of 1.2 W cm^−2^. (p) Loading efficiency of siFn1 at various weight ratios of BiO_2‐X_ to siFn1. Data were expressed as means ± SD. ^*^ *p* < 0.05, ^**^ *p* < 0.01, ^***^ *p* < 0.001, ^****^ *p* < 0.0001, n.s., no significance.

Accordingly, when O_2_ was lost in the [BiO_2_]^−^ from NaBiO_3_ starting material, abundant oxygen vacancies are generated in BiO_2−X_ NSs. As depicted in Figure 3c, the transmission electron microscopy (TEM) examination before ultrasonic fragmentation showed that the BiO_2−X_ NPs possessed a compact and relatively bulky morphology. Ultrasonic treatment substantially decreased the thickness of the BiO_2−X_ NSs, which displayed a lateral size of about 323.3 ± 73.09 nm, consistent with an ultrathin nanosheet morphology (Figure 3d,e). As shown in Figure 3f, all diffraction peaks of BiO_2−X_ were well indexed to its cubic crystalline phase (JCPDS No. 47–1057). Relative to the BiO_2−X_ NPs, the BiO_2−X_ NSs showed slightly weakened diffraction intensities and minor peak shifts, which may be attributed to the increased concentration of oxygen vacancies generated during ultrasonic fragmentation. Figure 3g shows that both spectra possess an evident signal at *g* = 2.004, a characteristic hallmark of oxygen vacancies. The electron paramagnetic resonance (EPR) signal intensity of BiO_2−X_ NPs is strengthened following exfoliation, further confirming the formation of additional oxygen vacancies in BiO_2−X_ NSs. The thickness of the as‐prepared BiO_2−X_ NSs was characterized by atomic force microscopy (AFM), confirming their ultrathin feature of BiO_2−X_ NSs with an average thickness of 3–4 nm (Figure 3h,i and Figure S1f).

Subsequently, the mPEG‐PEI‐modified BiO_2−X_ NSs were used to load siFn1 (BiO_2−X_/siFn1) through electrostatic interaction. The mean zeta potential of BiO_2−X_@PP was 6.86 mV, and the mean zeta potential of the final BiO_2−X_/siFn1 was determined to be 3.12 mV (Figure 3j). According to the results of the potential test, the successful loading of siFn1 was illustrated. The corresponding element mapping reveals that bismuth and oxygen elements are present homogeneously in the BiO_2−X_; phosphorus (P) was added to the nanosheets after loading siFn1 (Figure 3k). Although faint colored signals corresponding to phosphorus could be observed in the energy‐dispersive X‐ray spectroscopy (EDS) mapping of the BiO_2−X_ group, the characteristic P Kα peak was indistinct in the matching EDS spectrum (Figure S1g), with peak intensity nearly equivalent to the background level. Moreover, this energy window was subject to interference from the tail of intense Bi M‐series peaks and background fitting artifacts. Accordingly, the P signal detected in the elemental mapping most likely arose from low‐count statistical noise, spectral peak overlap, or artificial amplification induced by automatic software normalization, which cannot corroborate the intrinsic existence of elemental phosphorus within the sample. By contrast, distinct characteristic peaks assigned to P were detected in the EDS spectrum of BiO_2−X_/siFn1, further verifying the successful loading of siFn1 onto the nanomaterial (Figure S1h).

The sonothermal characteristics of the delivery nanosystem were evaluated following US irradiation. The temperature variation of BiO_2−X_ is presented in Figure 3l, which suggests that the sonothermal performance increased with BiO_2−X_ concentration. After 3 min US irradiation, the temperature of BiO_2−X_ with a concentration of 50 µg mL^−1^ increased to 44.5°C. In the control group without BiO_2−X_, the temperature only increased to 29°C. Moreover, no obvious temperature elevation of water was observed under various US power, ruling out the interference originating from the solvent (Figure S1i,j). These results further verify the sonothermal performance of the as‐prepared material itself.

Furthermore, the heating behavior of the nanosystem was evaluated at different US power densities (Figure 3m). The results demonstrated that the nanosystem produced an evident heating effect under various US intensities, thereby highlighting the controllable sonothermal performance of the BiO_2−X_ delivery system. The corresponding thermal images of BiO_2−X_ are shown in Figure 3n. It shows that the sonothermal effect of the system has the time and power dependence of ultrasonic irradiation. In addition, US irradiated 150 s and turned off US and cooled to room temperature for 3 cycles to detect and record temperature changes to study sonothermal stabilization (Figure 3o). No obvious alterations in sonothermal performance were observed during these periods. These results indicated that the BiO_2−X_ delivery system may serve to eradicate tumor cells via sonothermal therapy. In addition, the mild sonothermal effect can loosen collagen, reduce tumor hardness, and build an environment that is not conducive to tumor growth [30].

The ability of BiO_2−X_ to bind and retain siFn1 was evaluated using gel electrophoresis. Based on the efficient siFn1 loading observed at a BiO_2−X_‐to‐siFn1 weight ratio of 37.5, this formulation was used in subsequent experiments (Figure 3p). We first assessed the gene silencing efficiency of BiO_2−X_/siFn1 complexes at various temperatures. The results revealed that BiO_2−X_/siFn1 exhibited robust gene silencing activity at 45°C, and retained appreciable silencing capacity even at 50°C (Figure S2a,b).

Agarose gel electrophoresis showed that no free siFn1 bands were detected from BiO_2−X_/siFn1 after treatment at 37°C–50°C, whereas a distinct band was observed following heparin displacement, indicating good siFn1‐loading stability at elevated temperatures (Figure S2c). Subsequently, the long‐term loading stability of BiO_2−X_/siFn1 was evaluated under different thermal conditions. The cumulative release of loaded siRNA remained low within 48 h. Although a slight elevation in siRNA release was observed at 50°C, the total cumulative release over 48 h was still less than 10%, demonstrating that the majority of siRNA was stably immobilized on the nanocarrier (Figure S2d). Collectively, these data verified the favorable siRNA loading stability and functional durability of BiO_2−X_/siFn1. RNase degradation protection assays further confirmed that BiO_2−X_ nanosheets effectively shielded encapsulated siRNA from nuclease‐mediated cleavage (Figure S2e). We next characterized the gene silencing performance of BiO_2−X_/siFn1 in diverse physiological solutions. Satisfactory silencing efficacy was maintained in phosphate‐buffered saline (PBS), 0.9% NaCl solution, and 10% fetal bovine serum (FBS) (Figure S2f,g). The above experimental results show that the BiO_2‐X_/siFn1 nano‐delivery system has been successfully prepared, and it exhibits favorable stability and a controllable sonothermal effect.

1,3‐diphenylisobenzofuran (DPBF) was used as a reactive oxygen species (ROS) indicator. DPBF alone, DPBF + BiO_2‐X_, and DPBF + BiO_2‐X_/siFn1 were subjected to US irradiation for different durations (0, 1, 2, and 3 min, 1.0 W cm^−2^). The absorbance at 410 nm progressively declined as the US irradiation duration increased, suggesting continuous ROS generation (Figure S2h–k). Compared with the DPBF‐only group, the DPBF + BiO_2‐X_ and DPBF + BiO_2‐X_/siFn1 groups exhibited significantly higher DPBF degradation rates, suggesting enhanced ROS generation in the presence of the BiO_2‐X_ nanosheets. These results indicate that the two‐dimensional nanosheets participate in and amplify US cavitation‐associated sonochemical effects.

### 2.3 Antitumor Performance of BiO_2‐X_/siFn1 + US Against HCC In Vitro

After successfully constructing the BiO_2‐X_/siFn1 system, we first verified its biosafety at the cellular level. Hepa1‐6 cells were incubated with different concentrations of BiO_2‐X_ for 24 or 48 h, and the results showed that BiO_2‐X_ had satisfactory biosafety (Figure 4a). To achieve siFn1‐induced gene silencing, nanodelivery systems should permit siFn1 to escape from endosomes and be released into the cytoplasm [31, 32]. We used confocal laser scanning microscopy (CLSM) to observe the localization of BiO_2‐X_/siFn1 nanosheets in organelles. US irradiation facilitated the dissociation of red fluorescence from cyanine 5.5 (Cy5.5)‐labeled BiO_2‐X_/siFn1 and green fluorescence from lysosomes. Pearson correlation analysis of the fluorescence images further indicated that BiO_2‐X_/siFn1 + US enabled efficient lysosomal escape (Figure 4b).

**FIGURE 4:**
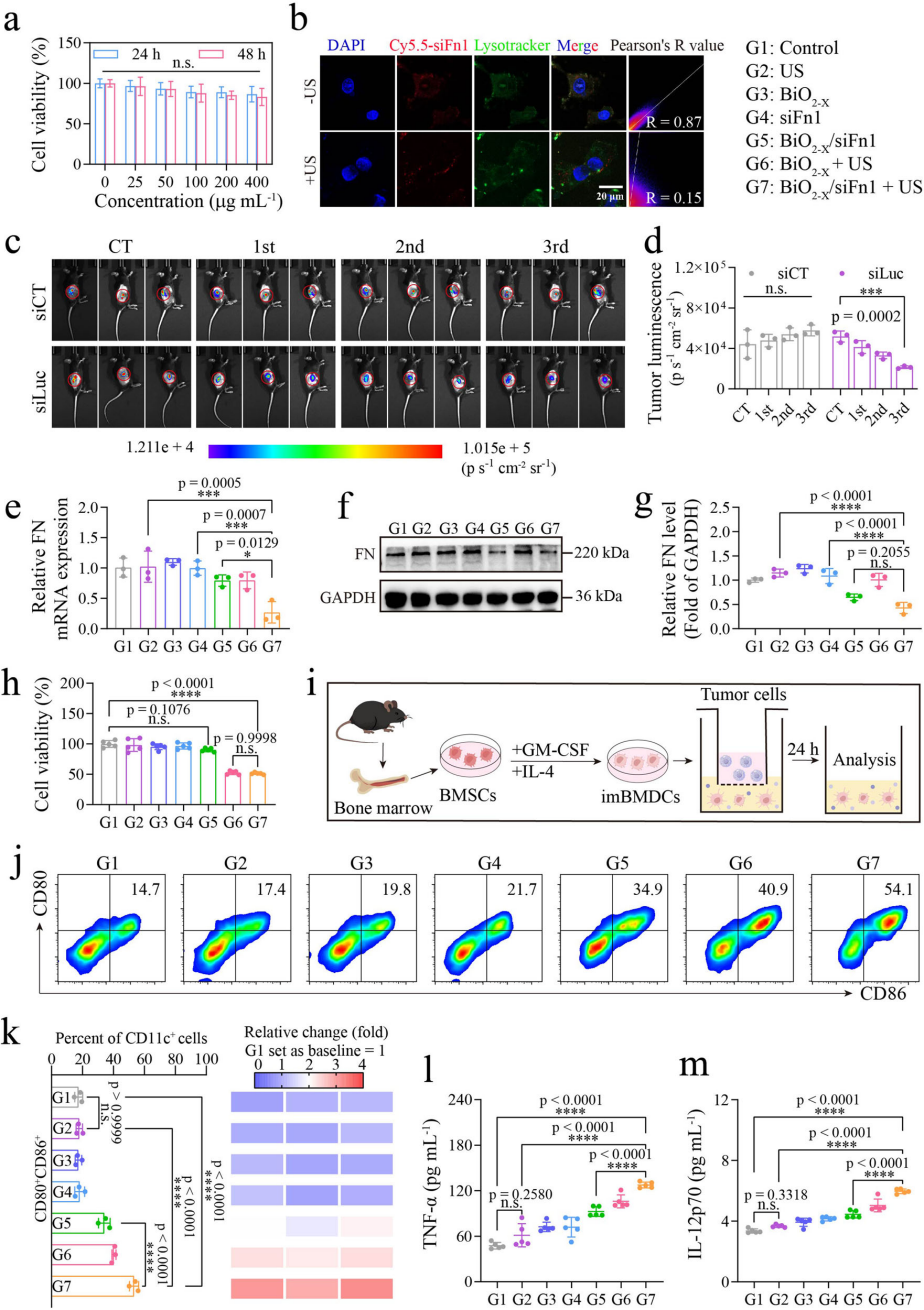
In vitro therapeutic efficacy of BiO_2‐X_/siFn1 against HCC. (a) Assessment of Hepa1‐6 tumor cell viability after co‐incubation with BiO_2‐X_ NSs for 24 and 48 h (BiO_2‐X_ = 0, 25, 50, 100, 200, and 400 µg mL^−1^) at different concentrations (*n* = 5). (b) Representative CLSM images and associated confocal analysis of Hepa1‐6 cells co‐labeled with LysoTracker Green and Cy5.5‐conjugated BiO_2‐X_/siFn1 in the presence or absence of US. R indicates the Pearson's correlation coefficient. (c) Tumor‐bearing C57BL/6 mice implanted with Hepa1‐6‐Luc cells received intravenous injections of BiO_2‐X_/siLuc every 5 days, with whole‐body bioluminescence imaging performed 24 h after each administration (*n* = 3). (d) Quantitative analysis of bioluminescence in vivo (*n* = 3). (e) Fn1 mRNA expression was quantified using quantitative real‐time polymerase chain reaction (qRT‐PCR) (*n* = 3). (f) FN protein expression in Hepa1‐6 cells after various treatments was evaluated by western blotting. (g) Quantitation of FN in western blotting (*n* = 3). (h) Hepa1‐6 tumor cell viability was assessed under the different treatment conditions (*n* = 5). (i) Diagrammatic illustration of the experimental setup for assessing DC maturation. Hepa1‐6 tumor cells in the upper chamber were subjected to different treatment regimens, whereas the lower chamber was used for BMDCs culture. (j) Typical flow cytometric profiles and (k) the corresponding statistical analysis of DCs (*n* = 3). (l) Secretion of TNF‐α and (m) IL‐12p70 from the supernatant of BMDCs (*n* = 5). Significance between multiple groups was calculated using one‐way analysis of variance (ANOVA), followed by Tukey's post hoc test for multiple pairwise comparisons when the overall ANOVA showed statistical significance. Control (G1), US (G2), BiO_2‐X_ (G3), siFn1 (G4), BiO_2‐X_/siFn1 (G5), BiO_2‐X_ + US (G6), and BiO_2‐X_/siFn1 + US (G7). Data were expressed as means ± SD. ^*^ *p* < 0.05, ^**^ *p* < 0.01, ^***^ *p* < 0.001, ^****^ *p* < 0.0001, n.s., no significance.

We examined the phagocytosis of BiO_2‐X_/siFn1 by cancer‐associated fibroblast (CAFs) cell lines. The results showed that, similar to Hepa1‐6 cells, BiO_2‐X_/siFn1 could also be successfully phagocytosed by NIH‐3T3 cells, indicating that BiO_2‐X_/siFn1 exerts its function in the NIH‐3T3 cell line as well (Figure S3a). To better understand the effect induced by US combined with siFn1, we used CLSM to observe the uptake of Cy5.5‐siFn1 by Hepa1‐6 cells. No obvious intracellular Cy5.5‐siFn1 fluorescence signal was observed in Hepa1‐6 cells, indicating that free siRNA was not markedly taken up by Hepa1‐6 cells. Similarly, no obvious Cy5.5‐siFn1 fluorescence signal was detected in the US + siFn1 group, suggesting that, under the US parameters used in this study, US treatment did not significantly promote the uptake of free siRNA by Hepa1‐6 cells (Figure S3b).

We subsequently evaluated the gene silencing efficiency of these BiO_2‐X_@PP by detecting luciferase (Luc) expression in Hepa1‐6‐Luc cells after corresponding treatment [33]. After treatment with BiO_2‐X_/Luc‐targeting siRNA (BiO_2‐X_/siLuc), the signal was significantly reduced and even did not show bioluminescence (Figure S3c,d). In vivo administration of siLuc resulted in a progressive reduction in Luc activity in Hepa1‐6‐Luc tumor‐bearing mice following three repeated injections (Figure 4c,d). It shows that BiO_2‐X_ as a carrier to deliver siRNA can achieve good gene silencing effect.

The cells were then exposed to BiO_2‐X_‐loaded siFn1, and the expression levels of Fn1 mRNA and FN protein were subsequently determined. The Fn1 mRNA and FN protein expression levels in the US‐only and free siFn1 groups were comparable to those in the control group. Compared with BiO_2‐X_/siFn1 treatment, BiO_2‐X_/siFn1 + US treatment further reduced Fn1 mRNA expression (*p* = 0.0129), whereas the difference in FN protein expression between the two groups did not reach statistical significance (*p* = 0.2055) (Figure 4e–g). FN protein and Fn1 mRNA expression levels were markedly decreased following treatment with BiO_2‐X_/siFn1 + US (Figure 4e–g). These results indicated that the BiO_2‐X_/siFn1 + US treatments decreased FN expression. Compared with the negative control (NC) messenger RNA group, US + siFn1 treatment did not significantly reduce FN protein expression in Hepa1‐6 cells, suggesting that US treatment failed to effectively promote free siFn1‐mediated FN knockdown at the protein level (Figure S3e,f).

The cell survival rate after different treatments was detected by Cell Counting Kit‐8 method. Treatment with either the BiO_2−X_ group alone or the BiO_2−X_/siFn1 group exerted no significant effects on the viability of tumor cells. The cell survival rate of BiO_2−X_ and BiO_2−X_/siFn1 decreased significantly after US irradiation, demonstrating that the nanosystem played a good sonothermal therapeutic performance (Figure 4h). Subsequently, calcein acetoxymethyl ester (Calcein‐AM) and propidium iodide (PI) staining (Figure S4a,b) were used for staining. The results further illustrate the tumor cell killing ability of the BiO_2−X_/siFn1 + US nanosystem.

To further determine whether BiO_2−X_/siFn1 + US treatment induced immunogenic cell death (ICD) in tumor cells, flow cytometry analysis and enzyme‐linked immunosorbent assay (ELISA) further confirmed that calreticulin (CRT) exposure on the tumor cell plasma membrane and high‐mobility group box 1 protein (HMGB1) secretion was significantly increased in the BiO_2−X_/siFn1 + US group (Figure S5a–c). These findings indicate that BiO_2−X_/siFn1 + US treatment can effectively induce ICD in tumor cells in vitro.

In order to further detect whether tumor cells undergoing ICD can elicit the antitumor immune response, we first detected whether BiO_2−X_/siFn1 + US treatment promoted the maturation of dendritic cells (DCs). Following maturation, DCs acquire migratory capacity and relocate to lymph nodes, where they initiate antigen presentation to naïve T lymphocytes [34, 35, 36]. On the basis of this mechanism, we used the Transwell system to detect the maturation of DCs to simulate these developmental processes (Figure 4i). Bone marrow–derived dendritic cells (BMDCs) from juvenile mice were isolated from bone marrow for subsequent experiments [37]. After 24 h of incubation, the BiO_2−X_ nanosystem, when combined with US irradiation, significantly induced DC maturation, whereas US exposure alone failed to elicit a comparable response. Compared with the BiO_2−X_/siFn1 group, conditioned medium from tumor cells treated with BiO_2−X_/siFn1 + US further increased the proportion of mature DCs, suggesting that the sonothermal and related effects generated by US activation of the BiO_2−X_ nanosystem enhance tumor cell‐mediated DC maturation (Figure 4j,k and Figure S5d).

The ELISA analysis revealed a significant upregulation of interleukin‐12p70 (IL‐12p70) and tumor necrosis factor‐α (TNF‐α) secretion from BMDCs following treatment with the BiO_2−X_ nanosystem under US irradiation (Figure 4l,m). US treatment alone did not significantly alter the levels of IL‐12p70 or TNF‐α. Compared with the BiO_2−X_/siFn1 group, the BiO_2−X_/siFn1 + US group significantly increased the secretion of IL‐12p70 and TNF‐α, further indicating that the sonothermal and related effects generated by US activation of the BiO_2−X_ nanosystem enhance tumor cell‐mediated DC maturation and functional activation (Figure 4l,m). These findings demonstrated that conditioned medium derived from Hepa1‐6 cells treated with the US‐irradiated BiO_2‐X_ nanosystem facilitated the maturation of DCs, thereby providing a basis for subsequent T‐cell activation.

### 2.4 ECM Remodeling and Reverse Anoikis Resistance

Since the resistance of PD‐1 antibody therapy is related to ECM deposition, it was further explored whether the treatment system can reshape ECM by acoustic heat and increase the extent of immune cell infiltration at the tumor site. After BiO_2‐X_/siFn1 + US treatment, the content of collagen‐I around the cells decreased significantly (Figure 5a,b). It is proved that the sonothermal effect can soften the ECM barrier. Pericellular collagen‐I deposition remained unchanged after US treatment alone compared with the control. There was no statistically significant difference between the BiO_2‐X_/siFn1 + US and BiO_2‐X_ + US groups. The value observed in the BiO_2‐X_/siFn1 group was also comparable to that in the control group. These results suggest that, under the present experimental conditions, siFn1‐mediated downregulation of FN expression did not induce a detectable change in type collagen‐ I deposition (Figure 5a,b).

**FIGURE 5:**
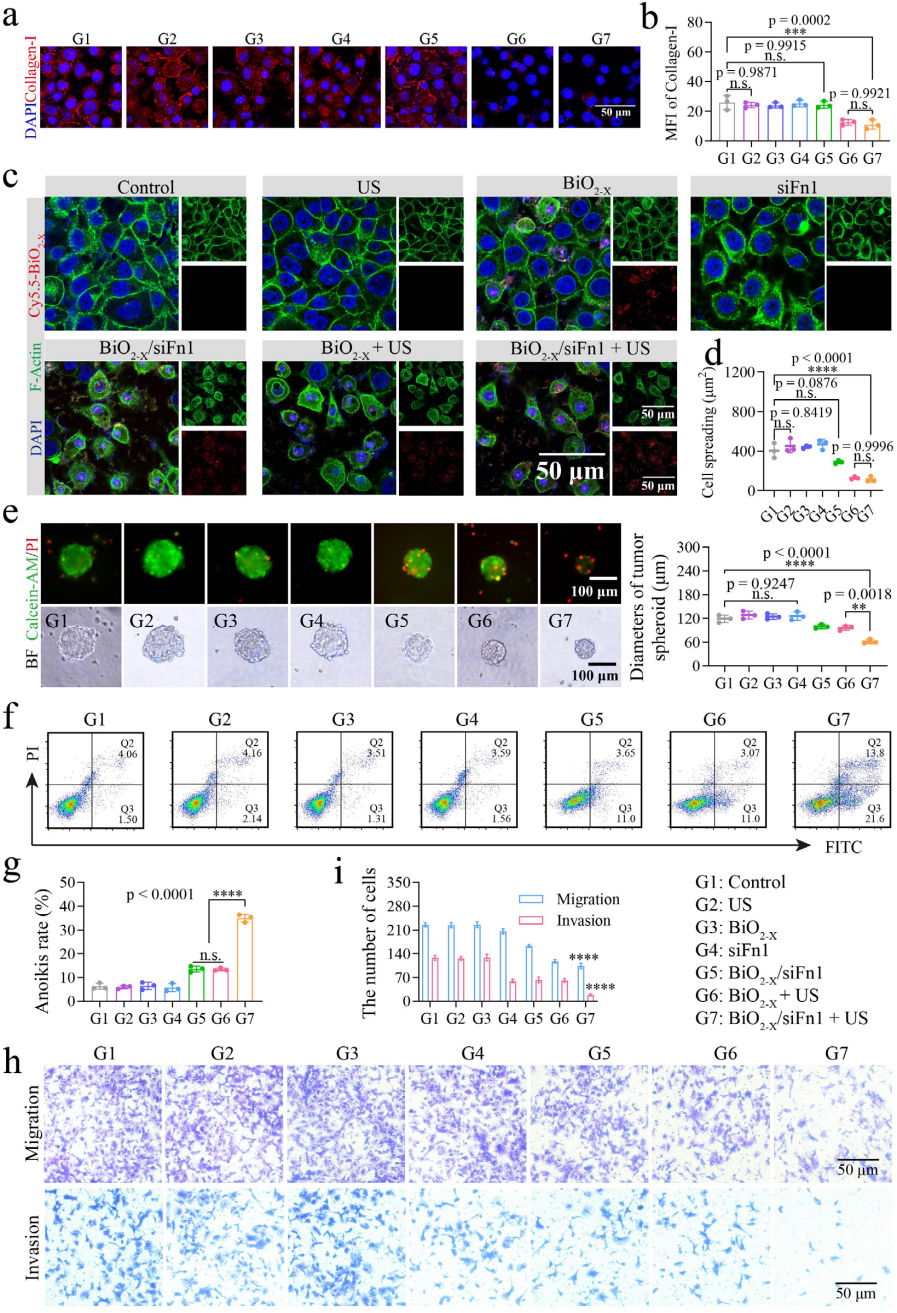
ECM remodeling and reverse anoikis resistance. (a) Collagen‐I expression of Hepa1‐6 cells by BiO_2‐X_/siFn1 + US. (b) The corresponding quantification of collagen‐I mean fluorescence intensity (MFI) (Alexa Fluor 555, red) using ImageJ (*n* = 3). (c) Assessment of cell morphology and intracellular internalization in Hepa1‐6 cells following exposure to Cy5.5‐BiO_2‐X_ for 4 h. (d) Cell spreading areas after collagen‐I degradation were quantitatively measured using the CLSM images in (c) (*n* = 3). (e) Fluorescence micrographs of Calcein‐AM/PI co‐stained 3D multicellular spheroids of Hepa1‐6 cancer cells, along with photo‐microscopy images and corresponding quantitative analysis of spheroid diameters (*n* = 3). (f) Anoikis was assessed by flow cytometry assay in Hepa1‐6‐AR treated with different groups. (g) Statistical data for apoptosis (*n* = 3). (h) Representative images of transwell migration and invasion assay for Hepa1‐6 treated with different groups. (i) Quantification results of transwell migration and invasion assay (*n* = 3). Significance between multiple groups was calculated using one‐way ANOVA, followed by Tukey's post hoc test for multiple pairwise comparisons when the overall ANOVA showed statistical significance. Control (G1), US (G2), BiO_2‐X_ (G3), siFn1 (G4), BiO_2‐X_/siFn1 (G5), BiO_2‐X_ + US (G6), and BiO_2‐X_/siFn1 + US (G7). Data were expressed as means ± SD. ^*^ *p* < 0.05, ^**^ *p* < 0.01, ^***^ *p* < 0.001, ^****^ *p* < 0.0001, n.s., no significance.

ECM can provide physical support for the tumor cytoskeleton, thereby helping cells maintain the rigid structure required for their invasive growth. After BiO_2‐X_/siFn1 + US treatment, the spreading area of Hepa1‐6 decreased significantly, which further indicated that ECM had mechanical softening (Figure 5c,d). Compared with the control group, US treatment alone did not significantly alter the spreading area of Hepa1‐6 cells. BiO_2‐X_/siFn1 treatment resulted in a decreasing trend in cell spreading area, although the difference did not reach statistical significance (*p* = 0.0876). No significant difference was observed between the BiO_2‐X_/siFn1 + US and BiO_2‐X_ + US groups, suggesting that the reduction in cell spreading area was primarily attributable to the sonothermal effects generated by US irradiation of the BiO_2‐X_ nanosystem (Figure 5c,d).

Tumor cells are characterized by anoikis resistance, and a loose ECM is crucial for the metastasis of primary tumor cells [38]. The cleverness of BiO_2‐X_/siFn1 combined with US is that siFn1 can reduce the expression of FN and reverse the anoikis resistance of tumor cells through integrin and other pathways. Subsequently, Hepa1‐6 cells treated with different methods were cultured in suspension for 7 days. It was observed that the cells in the BiO_2‐X_/siFn1 + US group had significantly higher cell death rates than the control group under matrix detachment conditions, and the tumor spheres formed were smaller in diameter, indicating that the treatment impaired tumor cell survival and expansion following loss of ECM adhesion and supported the suppression of the anoikis‐resistant phenotype (Figure 5e). Compared with the control group, free siFn1 treatment did not significantly alter tumor spheroid diameter, suggesting that free siFn1 was unable to affect the resistance of tumor cells to anoikis. Compared with the BiO_2‐X_ + US group, the BiO_2‐X_/siFn1 + US group exhibited a further reduction in tumor spheroid diameter, suggesting that siFn1‐mediated downregulation of FN expression further inhibited tumor cell survival and expansion under non‐adherent conditions and may have weakened their resistance to anoikis (Figure 5e).

To further determine the impact of various treatments on the anoikis resistance phenotype of tumor cells, Hepa1‐6 cells with anoikis resistance (Hepa1‐6‐AR) were treated with the above different groups, and then continued suspension culture. Cells were harvested for flow cytometric analysis, and the findings indicated that the anoikis rate of Hepa1‐6‐AR cells was significantly elevated in the BiO_2‐X_/siFn1 + US treatment group compared with the BiO_2‐X_ + US group, suggesting that BiO_2‐X_/siFn1 + US partially reversed the resistance of tumor cells to anoikis (Figure 5f,g). The anoikis resistance of tumor cells can lead to metastasis, so the invasion and migration ability of Hepa1‐6 cells after different treatments was further detected [39, 40]. Transwell assay indicated that BiO_2‐X_/siFn1 + US significantly decreased the migration and the invasion capacity of Hepa1‐6 cells (Figure 5h,i). In conclusion, BiO_2‐X_/siFn1 + US could remodel the ECM through sonothermal effects while reversing the anoikis resistance of tumor cells, thereby reducing their invasion and migration capabilities.

### 2.5 Effectiveness of BiO_2‐X_/siFn1 + US In Vivo

Based on the promising in vitro findings, the efficacy of BiO_2‐X_/siFn1 + US therapeutic effect in vivo was evaluated. Initially, the hemocompatibility of BiO_2‐X_/siFn1 was assessed. The findings revealed that the hemolysis rate of BiO_2‐X_/siFn1 after incubation with plasma was below 0.2% (Figure S6a,b), which were below the internationally accepted threshold of 5% [41]. Next, the major organs of the mice were extracted for hematoxylin‐eosin (HE) staining, showing a physiological morphology similar to the level recorded in mice before injection (Figure S6c). Moreover, the biochemical indicators of blood, liver, and kidney function in the experimental mice did not differ significantly from those observed in the control group (Figures S6d and S7a).

To evaluate the potential metal ion‐related toxicity of the nanocarrier BiO_2‐X_, inductively coupled plasma mass spectrometry (ICP‐MS) was used to analyze digested organ tissues and excretion samples, including urine and feces. This analysis was performed to track the in vivo distribution and clearance of bismuth (Bi) in major organs within 30 days after administration (Figure S7b–d). Bi signals showed different distribution patterns among the collected organs. The Bi signal was mainly detected in the liver, spleen, and kidney, whereas the Bi content in the heart and lung remained relatively low. Notably, by day 30, the Bi content in all examined organs had decreased to the baseline level of the blank control group, namely PBS‐treated mice, indicating that these nanoparticles caused almost no Bi accumulation in vivo during the 30‐day observation period (Figure S7b). In urine and fecal samples, the Bi content reached its highest level on day 1 and then rapidly decreased. By day 30, the Bi levels in both urine and feces were also close to those in the PBS group. These results indicate that BiO_2‐X_ could be gradually cleared from the body and excreted through both urinary and fecal pathways, suggesting a low risk of long‐term metal element accumulation. These findings demonstrate the favorable biosafety and biocompatibility of the BiO_2‐X_/siFn1 + US nanosystem, supporting its further evaluation in subsequent in vivo experiments.

To begin with, the in vivo accumulation behavior of BiO_2‐X_/siFn1 was investigated using an in vivo imaging system. The findings revealed a pronounced enrichment of BiO_2‐X_/siFn1 in the tumor tissue (Figure 6a,b). Further ex vivo imaging demonstrated preferential accumulation of BiO_2‐X_/siFn1 in tumor tissue, as evidenced by a markedly higher signal than that of Cy5.5‐siFn1 or free dye, while negligible signals were detected in other major tissues (Figure S8a,b).

**FIGURE 6:**
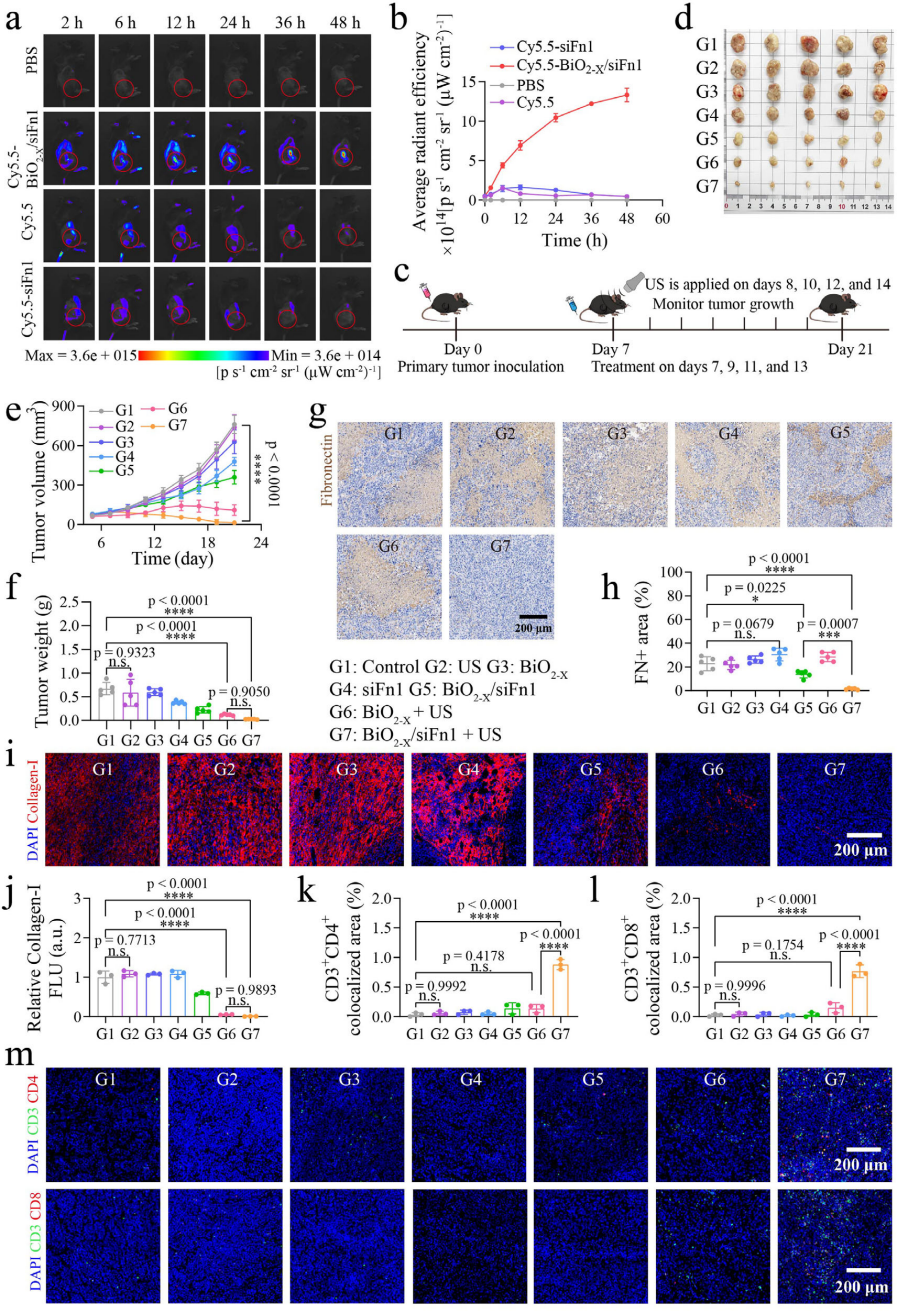
Effectiveness of BiO_2‐X_/siFn1 + US in vivo. (a) In vivo imaging of Cy5.5‐labeled BiO_2‐X_ NSs, Cy5.5‐labeled siFn1, and Cy5.5 in mice. (b) The average fluorescence signal in mice was quantitatively evaluated at different time points after the indicated treatments (*n* = 3). (c) Schematic diagram of experimental plan. (d) Photographic images of dissected tumor tissues collected from different treatment groups. (e) Variation in tumor volume among mice in the various administration groups (*n* = 10). (f) Quantitative comparison of excised tumor weights among different treatment groups (*n* = 5). (g) Representative images of tumor IHC for FN in mice at day 21. (h) Quantification of FN‐stained area in tumors from mice in the various experimental groups (*n* = 5). (i) IF images of collagen‐I from tumor tissue. The cell nuclei were stained with DAPI and are presented in blue. (j) Quantitative analysis of IF images (*n* = 3). (k) Quantification of CD3^+^/CD4^+^ colocalized pixels within the total area (*n* = 3). (l) The percentage of CD3^+^/CD8^+^ colocalized pixels within the total area was quantitatively assessed (*n* = 3). (m) IF images showing CD3^+^CD4^+^ and CD3^+^CD8^+^ cells in primary Hepa1‐6 tumor tissue sections. For panel (e), statistical differences were assessed by two‐way ANOVA with Geisser–Greenhouse correction, followed by Tukey's multiple comparisons test for post hoc analysis to determine statistical significance; statistical analyses for panels (f, h, j–l) were performed using one‐way ANOVA, followed by Tukey's post hoc test for multiple pairwise comparisons when the overall ANOVA showed statistical significance. Control (G1), US (G2), BiO_2‐X_ (G3), siFn1 (G4), BiO_2‐X_/siFn1 (G5), BiO_2‐X_ + US (G6), and BiO_2‐X_/siFn1 + US (G7). Data were expressed as means ± SD. ^*^ *p* < 0.05, ^**^ *p* < 0.01, ^***^ *p* < 0.001, ^****^ *p* < 0.0001, n.s., no significance.

The in vitro results showed that US + siFn1 treatment did not significantly reduce FN protein expression in Hepa1‐6 cells. To further evaluate the in vivo antitumor effect of this treatment, as shown in Figure S8c, tumor‐bearing mice received siFn1 via tail‐vein injection every 2 days for a total of four administrations, and tumor growth was monitored throughout the treatment period. The results showed that tumors in the PBS, US, siFn1, and siFn1 + US groups all exhibited rapid growth, and the siFn1 + US group did not show significant inhibition of tumor growth compared with the PBS group (Figure S8d,e). These findings indicate that US + siFn1 failed to produce an effective in vivo antitumor response. Therefore, this treatment strategy was not investigated further in the subsequent experiments.

We next examined the antitumor efficacy of BiO_2‐X_/siFn1 + US in vivo. As outlined in Figure 6c, the mice received intravenous injections of the indicated drugs at 2‐day intervals, with four administrations in total. To further determine the antitumor efficacy of BiO_2‐X_/siFn1 in combination with US, all animals were sacrificed on day 21, followed by tumor excision. Representative tumor images are shown in Figure 6d, which demonstrate that BiO_2‐X_/siFn1 + US treatment markedly suppressed tumor growth. As depicted in Figure 6e and Figure S8f, rapid tumor growth was observed in the PBS, US, BiO_2‐X_, siFn1, and BiO_2‐X_/siFn1 groups. Moreover, the BiO_2‐X_ + US group demonstrated a slower tumor growth rate, likely owing to the mild sonothermal effect. Importantly, the BiO_2‐X_/siFn1 + US treatment group demonstrated markedly greater antitumor efficacy than all other groups, and even achieved complete tumor ablation. This phenomenon is likely attributable to the combined effect of mild sonothermal effects and siFn1, which soften and loosen the ECM structure, allowing BiO_2‐X_/siFn1 to penetrate deeper into the tumor tissue and effectively eradicate the tumor. The measurement of tumor weight was consistent with the above results (Figure 6f). Notably, treated mice exhibited no significant body weight loss, and survival was markedly prolonged, confirming the outstanding biosafety and effective therapeutic performance of the BiO_2‐X_/siFn1 + US nanosystem (Figure S9a–c).

The results of FN IHC staining of tumor tissues showed that the BiO_2‐X_/siFn1 + US treatment group could achieve good silencing effect in vivo (Figure 6g,h). Compared with the control group, free siFn1 treatment resulted in a decreasing trend in the FN‐positive area within tumor tissues, although the difference did not reach statistical significance (*p* = 0.0679). In contrast, BiO_2‐X_/siFn1 treatment significantly reduced the FN‐positive area (*p* = 0.0225), indicating that the BiO_2‐X_ nanocarrier improved the in vivo delivery and gene‐silencing efficacy of siFn1. Compared with BiO_2‐X_/siFn1 alone, BiO_2‐X_/siFn1 + US further decreased the FN‐positive area (*p* = 0.0007). This enhanced effect may be attributed to sonothermal and associated effects that soften and loosen the tumor ECM, thereby reducing the barrier to intratumoral diffusion of the nanocarrier. This may facilitate the deeper penetration of BiO_2‐X_/siFn1 into tumor tissues and its uptake by tumor cells, ultimately enhancing siFn1‐mediated FN downregulation (Figure 6g,h).

In the BiO_2‐X_/siFn1 + US group, the ECM component of collagen‐I was severely loosened (Figure 6i,j). US exposure alone produced no significant change in the relative fluorescence intensity of collagen‐I compared with the control group, whereas BiO_2‐X_ combined with US significantly reduced the collagen‐I fluorescence signal. These results indicate that the reduction in the collagen‐I signal was not caused by US alone but mainly depended on the sonothermal and related effects generated by US irradiation of the BiO_2‐X_ nanosystem (Figure 6i,j). It shows that BiO_2‐X_/siFn1 + US also has obvious ECM remodeling effect in vivo.

To quantitatively evaluate the effects of BiO_2‐X_/siFn1 + US treatment on the mechanical properties of the tumor ECM, we measured the matrix stiffness of tumor tissues using bio‐AFM. To distinguish thermal effects from US‐associated mechanical effects, a BiO_2‐X_/siFn1 + heating control group was included. This group received a temperature–time profile matched to that of the BiO_2‐X_/siFn1 + US group but was not exposed to US. Bio‐AFM stiffness maps and distribution profiles revealed markedly lower matrix stiffness in tumor tissues from the BiO_2‐X_/siFn1 + US group than in those from the control group. Although the matrix stiffness was also reduced in the BiO_2‐X_/siFn1 + heating group, the extent of the reduction was markedly smaller than that observed in the BiO_2‐X_/siFn1 + US group (Figure S9d,e). These results indicate that temperature‐matched heating alone is insufficient to fully account for the matrix softening induced by BiO_2‐X_/siFn1 + US treatment, supporting the involvement of US‐associated nonthermal effects in altering the mechanical properties of the ECM.

IF staining of tumor tissue revealed a significant elevation in the infiltration of helper T lymphocytes and cytotoxic T lymphocytes within the tumor tissue (Figure 6k–m and Figure S10a,b). Taken together, these experimental results suggest that the nanosystem can kill tumor cells through the sonothermal effect under the action of US, silence Fn1 gene and reshape ECM, enhance immune cell infiltration at the tumor site, and show excellent anti‐tumor effect.

### 2.6 BiO_2‐X_/siFn1 + Us Therapy Elicits a Potent Immune Response In Vivo

We further explored whether the potent antitumor effects exerted by BiO_2‐X_/siFn1 upon US irradiation were attributable to immune activation. First, the levels of damage‐associated molecular patterns (DAMPs)‐related indicators were assessed. The results showed that the BiO_2‐X_/siFn1 + US‐treated group exhibited higher levels of cell membrane surface CRT fluorescence, along with decreased nuclear HMGB1 fluorescence intensity, indicating the most pronounced HMGB1 translocation and release. These results indicate that DAMPs are critical for DCs recruitment and maturation (Figure S11a–c). After treatment, tumors and spleens were harvested for analysis of antitumor immune cell populations and key immune‐related factor expression. The flow cytometry results demonstrated that the BiO_2‐X_/siFn1 combined with US treatment group exhibited a significantly greater proportion of mature DCs than the other groups, reaching an average of 32.4% ± 0.4% (Figure 7a and Figure S11d,e). Like in vitro studies, sonothermal therapy can significantly stimulate the maturation of DCs in tumor tissues in vivo.

**FIGURE 7:**
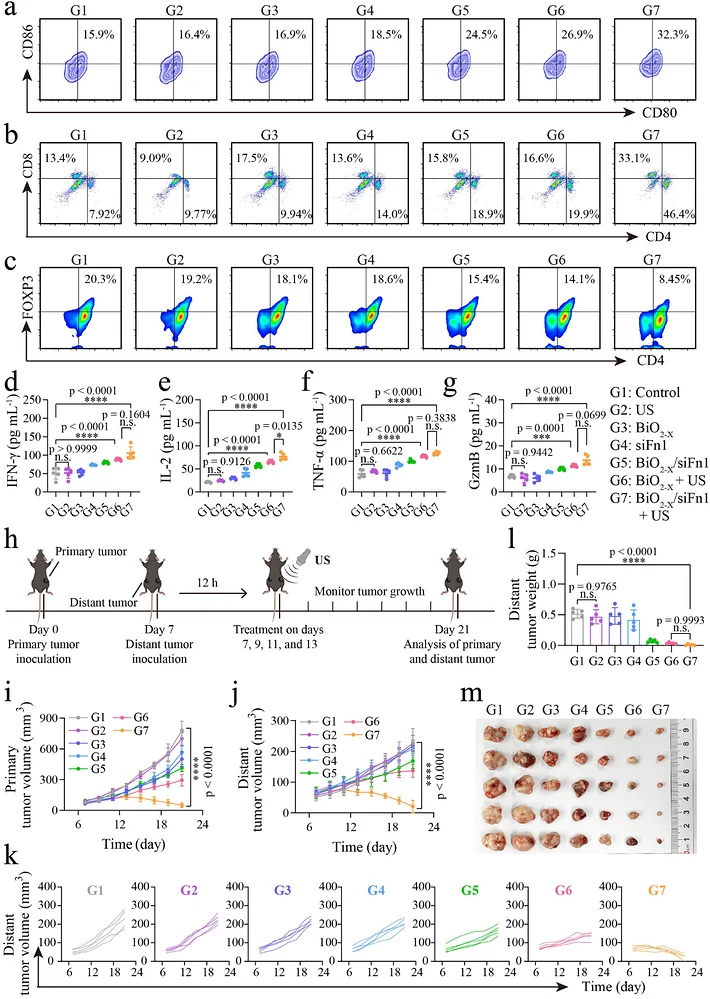
Immune activation and abscopal effect. (a) Representative flow cytometric analysis of mature DCs (CD80^+^CD86^+^). (b) Representative plots of CD4^+^ and CD8^+^ T cells in splenic tissue under different treatment conditions. (c) Typical flow cytometry of Treg cells (FOXP3^+^CD4^+^). (d) The level of IFN‐γ in the TME was used to evaluate the local immune response (*n* = 5). (e) The expression level of IL‐2 in tumor tissues after different treatments (*n* = 5). (f) The expression level of TNF‐α in the TME was used to evaluate the local immune status (*n* = 5). (g) GzmB concentration (*n* = 5). (h) Flow diagram of the experimental design for the in situ distal dual‐tumor model. (i) Mean growth profile of the primary tumor (*n* = 5). (j) Mean growth curves and (k) corresponding growth curves of distant tumors (*n* = 5). (l) Distal tumor weights in the various treatment groups (*n* = 5). (m) Gross images of primary tumors in different treatment groups. For panel (i, j), statistical differences were assessed by two‐way ANOVA with Geisser–Greenhouse correction, followed by Tukey's multiple comparisons test for post hoc analysis to determine statistical significance, whereas panels (d–g, l) were analyzed using one‐way ANOVA, followed by Tukey's post hoc test for multiple pairwise comparisons when the overall ANOVA showed statistical significance. Control (G1), US (G2), BiO_2‐X_ (G3), siFn1 (G4), BiO_2‐X_/siFn1 (G5), BiO_2‐X_ + US (G6), and BiO_2‐X_/siFn1 + US (G7). Data were expressed as means ± SD. ^*^ *p* < 0.05, ^**^ *p* < 0.01, ^***^ *p* < 0.001, ^****^ *p* < 0.0001, n.s., no significance.

In addition, after BiO_2‐X_/siFn1 + US treatment (Figure 7b and Figure S11f–i), a significant increase in the splenic population of cytotoxic T cells (CD8^+^ cells) was observed. At the same time, helper T cells (CD4^+^ cells) additionally elevated significantly. It indicates that the systemic immune response is activated. Compared with the BiO_2‐X_ + US group, the BiO_2‐X_/siFn1 + US group had more splenic T cells. The reason was that the BiO_2‐X_/siFn1 + US group had more DC maturation and activation to produce more T cells. Moreover, evaluation of regulatory CD45^+^CD3^+^CD4^+^FOXP3^+^ cells within the tumor tissue showed a significant decrease in regulatory T cell (Treg) standard deviation infiltration in the BiO_2‐X_/siFn1 + US group (Figure 7c and Figure S12a,b). This result indicates that the nanosystem improves the antitumor effect through the attenuation of immunosuppressive status. This indicates that the nanosystem can promote immune activation, inhibit the recruitment of Tregs and alleviate immunosuppression.

During the course of these antitumor immune responses, immune activation was assessed by measuring the concentrations of several characteristic cytokines, namely interleukin‐2 (IL‐2), TNF‐α, interferon‐γ (IFN‐γ), and granzyme B (GzmB). A considerable upregulation of these cytokines was observed in the BiO_2‐X_/siFn1 + US group (Figure 7d–g). IFN‐γ and GzmB serve as key effector mediators of cytotoxic T lymphocytes. Their elevated levels directly indicate the presence of functionally active effector immune cell infiltration and activation in the TME.

US exposure alone failed to significantly elevate IFN‐γ or GzmB levels relative to the control, whereas treatment with BiO_2‐X_ + US markedly enhanced the production of both factors. These results indicate that the increased secretion of IFN‐γ and GzmB was not induced by US alone but mainly depended on the sonothermal and related effects generated by US‐activated BiO_2‐X_ nanosystems. IL‐2 is a key factor in the proliferation and differentiation of T cells in tumor tissues (Figure 7e). The increase of IL‐2 level reflects the enhancement of local anti‐tumor immune response. The level of TNF‐α in the BiO_2‐X_/siFn1 + US group was significantly increased, indicating that the local immune response was enhanced. The TNF‐α level in the BiO_2‐X_/siFn1 + US group exhibited a markedly higher level than that in the BiO_2‐X_ + US group, as TNF‐α is primarily produced by mature DCs in the tumor tissue (Figure 7f). In summary, the results fully illustrate the immune activation effect caused by BiO_2‐X_/siFn1 + US treatment.

Splenic flow cytometry and tumor tissue IF reflect different stages of the antitumor immune response. The increased proportions of CD3^+^CD4^+^ and CD3^+^CD8^+^ T cells in the spleen primarily reflect systemic T‐cell activation and expansion induced by BiO_2‐X_/siFn1 + US treatment (Figure 7b and Figure S11f–i). In contrast, the accumulation of T cells within tumor tissues depends not only on the availability of peripheral effector T cells but also on the restriction imposed by the tumor ECM barrier on T‐cell migration and infiltration (Figure 6k–m, Figure S10a,b). The G7 group simultaneously increased both the pool of effector T cells available for recruitment and the accessibility of the tumor tissue, which may account for the nonlinear and synergistic increase in intratumoral T‐cell infiltration.

Treatment with G6 (BiO_2‐X_ + US) could induce a certain degree of tumor cell damage, DAMP release, DC maturation, and physical ECM remodeling, but lacked siFn1‐mediated FN downregulation. In contrast, treatment with G5 (BiO_2‐X_/siFn1) lacked US‐facilitated cytosolic delivery, immunogenic tumor cell damage, and US‐associated ECM remodeling. Treatment with G7 (BiO_2‐X_/siFn1 + US) simultaneously promoted DC maturation and T‐cell activation while weakening the tumor ECM barrier through the combined effects of physical matrix remodeling and Fn1 silencing. Consequently, a greater number of activated T cells were able to enter the tumor, resulting in substantially higher intratumoral T‐cell infiltration than that observed in either individual treatment group. This finding is more likely to reflect a synergistic effect between immune activation and local ECM barrier disruption. We also note that splenic flow cytometry and tumor IF were performed using different tissue sources and quantitative metrics; therefore, the magnitudes of the changes observed using these two methods should not be directly compared.

Furthermore, dual orthotopic and distal tumor model was employed to assess the effectiveness of the BiO_2‐X_/siFn1 + US therapeutic strategy in suppressing distal tumors [42]. Therefore, an in situ distal dual‐tumor model was established by subcutaneous inoculation of Hepa1‐6 cells into the right dorsal side of mice to produce the primary tumor, with an identical injection administered to the left side 7 days later to generate the distal tumor. When the primary tumor volume had increased to approximately 200–250 mm^3^, treatment was administered by tail vein injection. US irradiation was administered exclusively to the primary tumor, while the growth of tumors on both sides was monitored and the resulting data were analyzed on day 21 (Figure 7h). As indicated by the tumor growth curves, the BiO_2‐X_ + US and BiO_2‐X_/siFn1 + US groups exhibited significantly attenuated growth of primary tumors relative to the control group (Figure 7i and Figure S12c), whereas distal tumor growth was significantly inhibited in the BiO_2‐X_/siFn1 + US group compared with the control group (Figure 7j,k). After treatment, the mice were euthanized, and bilateral tumors were harvested and weighed. The findings were in agreement with the previous results, further demonstrating the favorable antitumor efficacy of BiO_2‐X_/siFn1 + US (Figure 7l,m and Figure S12d). Compared with the control group, US treatment alone failed to inhibit tumor growth, whereas BiO_2‐X_ + US markedly suppressed tumor growth (Figure 7l and Figure S12d). The mechanism underlying the suppression of distal tumors is likely associated with the broad activation of the immune system triggered by the BiO_2‐X_/siFn1 + US therapeutic regimen.

### 2.7 BiO_2‐X_/siFn1 + US Therapy Inhibits Lung Metastasis and Reverses Resistance to PD‐1 Antibody Therapy

FN regulates resistance to anoikis through the formation of cellular aggregates, thereby promoting tumor metastasis [13]. Subsequently, we evaluated the in vivo efficacy of BiO_2‐X_/siFn1 + US in suppressing pulmonary metastasis of tumors using C57BL/6 mouse models, as shown in Figure 8a. Furthermore, on day 42 following tumor implantation, mice in the BiO_2‐X_/siFn1 + US treatment group were sacrificed to evaluate lung metastasis [43]. Furthermore, lung tissues were collected, photographed, and subjected to HE staining. Mice treated with BiO_2‐X_/siFn1 + US exhibited significantly fewer pulmonary metastatic nodules than those in the other groups (Figure 8b,c).

**FIGURE 8:**
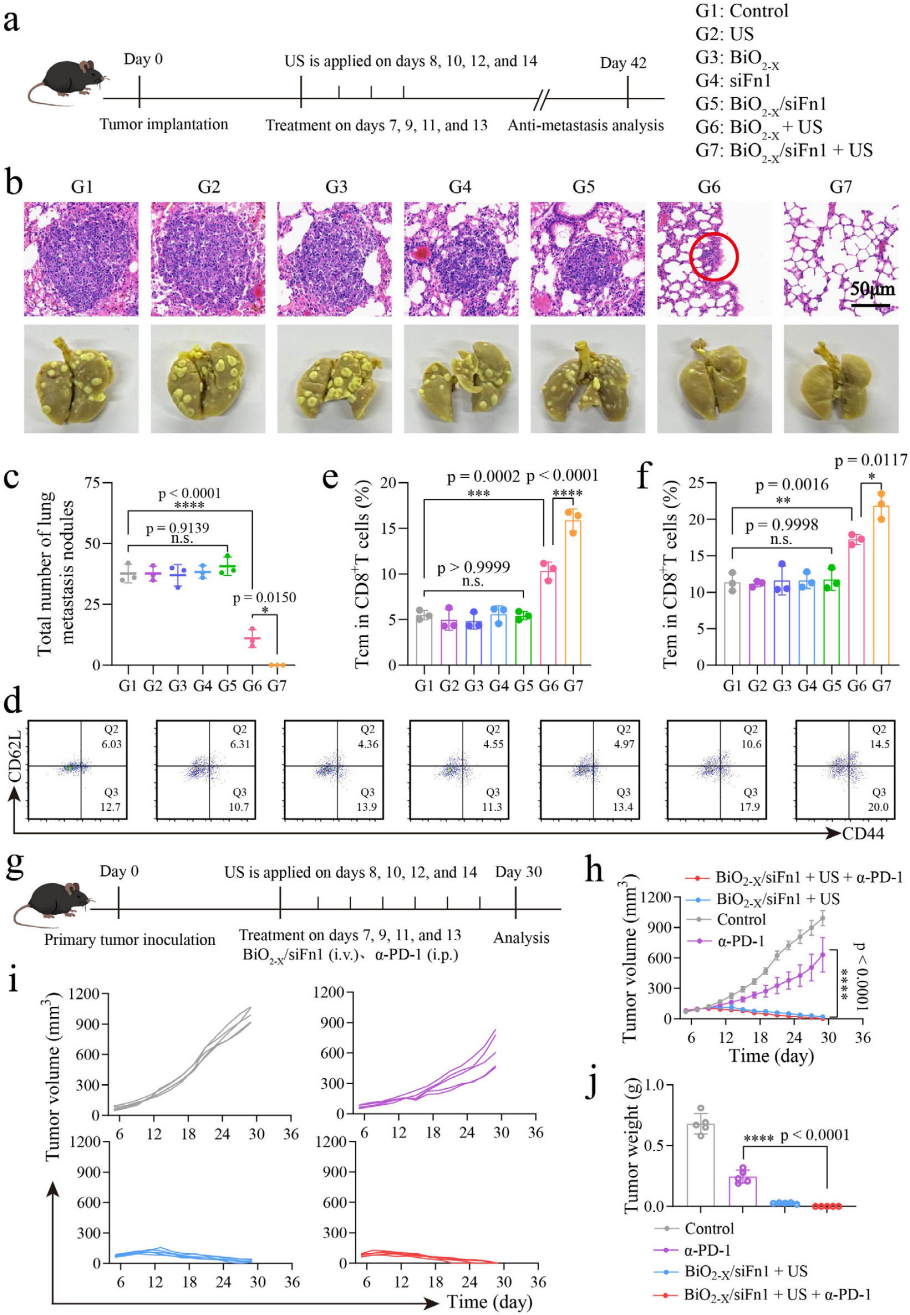
Inhibiting lung metastasis and combined with PD‐1 antibody therapy. (a) Diagrammatic representation of the in vivo experimental workflow using C57BL/6 mice for evaluating the anti‐metastatic effect of BiO_2‐X_/siFn1 + US. (b) Representative photographs and HE‐stained images of lungs with metastatic lesions. (c) Quantitative assessment of pulmonary metastatic nodules (*n* = 3). (d) Typical flow cytometry profiles of splenic effector memory T cells (Tem) and central memory T cells (Tcm) after various treatments. (e) Quantification of Tcm and (f) Tem in the spleen after various treatments (*n* = 3). (g) Schematic diagram of BiO_2‐X_/siFn1 + US combined with PD‐1 antibody treatment. (h) Mean tumor growth curves together with the (i) corresponding growth curves of tumors in each group (*n* = 5). (j) The weight of tumors in different treatment groups (*n* = 5). For panel (h), statistical differences were assessed by two‐way ANOVA with Geisser–Greenhouse correction, followed by Tukey's multiple comparisons test for post hoc analysis to determine statistical significance; statistical analyses for panels (c, e, f, j) were performed using one‐way ANOVA, followed by Tukey's post hoc test for multiple pairwise comparisons when the overall ANOVA showed statistical significance. Control (G1), US (G2), BiO_2‐X_ (G3), siFn1 (G4), BiO_2‐X_/siFn1 (G5), BiO_2‐X_ + US (G6), and BiO_2‐X_/siFn1 + US (G7). Data were expressed as means ± SD. ^*^ *p* < 0.05, ^**^ *p* < 0.01, ^***^ *p* < 0.001, ^****^ *p* < 0.0001, n.s., no significance.

Flow cytometry results demonstrated that the BiO_2‐X_/siFn1 + US treatment modality facilitated the induction of memory T cells, which contributed to durable antitumor effects and prevention of pulmonary metastasis (Figure 8d–f and Figure S13a). Subsequently, further evaluation was performed using BALB/c mouse models, and consistent results were obtained, demonstrating that BiO_2‐X_/siFn1 + US can effectively prevent pulmonary metastasis (Figure S13b–h).

We have proved through a large number of experiments that the treatment can dissolve ECM and reverse anoikis resistance through the sonothermal effect and activate the immune response in vivo to enhance the anti‐tumor effect. In order to verify whether the treatment plan can improve the problem of anti‐PD‐1 resistance in the clinical treatment of liver cancer, we once again combined with anti‐PD‐1 to treat mouse liver cancer model to explore. The therapeutic efficacy of combination therapy in HCC was evaluated using C57BL/6 mice. The treatment plan is depicted in Figure 8g. The volume of the tumor was recorded during the treatment and the tumor tissue was collected after the treatment. The results showed that compared with PD‐1 antibody monotherapy, the combination of BiO_2‐X_/siFn1 + US with PD‐1 antibody demonstrated a superior therapeutic effect and did not lead to the drug resistance associated with PD‐1 antibody monotherapy (Figure 8h–j).

## 3 Discussion

One of the main challenges of HCC immunotherapy is the physical and immune barriers in the TME [44], especially the dense ECM, which severely limits the penetration of therapeutic agents into tumor tissue and T lymphocytes infiltration [45, 46]. Accordingly, the progression of more efficient approaches to enhance immunotherapeutic efficacy and maximize its clinical utility is urgently needed [47].

In this study, by constructing a PD‐1 antibody‐resistant mouse tumor tissue model, it was directly proved that the ECM pathway‐related genes in PD‐1 antibody‐resistant tumor tissues were significantly enriched, and the up‐regulation of Fn1 gene expression mediated immunosuppressive microenvironment, which ultimately accelerated tumor progression [48]. US, as a non‐invasive, deeply tissue‐penetrating, and clinically widely used imaging and therapeutic tool, can be readily integrated with nanomaterials to facilitate clinical translation [49]. Therefore, this study developed a synergistic strategy based on BiO_2‐X_ combined with US irradiation and gene silencing (BiO_2‐X_/siFn1 + US), aiming to degrade ECM and target FN protein to reverse the resistance of immunotherapy, which provides a new idea for improving the effect of HCC immunotherapy.

To enhance the efficacy of immune checkpoint blockade therapy, previous studies have employed singlet oxygen (^1^O_2_) generated during sonodynamic processes to trigger the local release of immunomodulators within tumors [50]. In contrast, the present study was designed based on mechanisms associated with resistance to anti‐PD‐1 therapy. By delivering siFn1 to downregulate FN expression, our strategy specifically targets ECM‐associated mechanisms underlying anti‐PD‐1 resistance. Previous studies have demonstrated that oxygen‐vacancy‐rich BiO_2‐X_ nanosheets can directly suppress tumors through the synergistic effects of piezocatalytic, enzyme‐mimetic, and sonothermal therapies, with the main emphasis placed on the material properties and piezocatalytic mechanisms of BiO_2‐X_ [16]. Building on this established US‐responsive BiO_2‐X_ material, we constructed BiO_2‐X_ as an siFn1 delivery platform based on the transcriptomic characteristics of acquired anti‐PD‐1 resistance in HCC. This platform not only downregulates FN protein expression but also enables physical ECM remodeling and antitumor immune activation. In addition, previous studies have employed defect engineering to enhance sonocatalytic ROS generation and induce pyroptosis, thereby activating antitumor immunity [51]. Beyond immune activation, the present strategy physically remodels the ECM to alleviate the stromal barrier and create more favorable conditions for effector immune cells to infiltrate the tumor interior, thereby enhancing the efficacy of immunotherapy.

Our results showed that systemic administration of BiO_2‐X_/siFn1 to kill tumor cells under US irradiation significantly reversed the immunosuppressive TME of HCC, suppressed tumor progression, and ultimately extended the survival of HCC‐bearing mice with normal immune function. BiO_2‐X_/siFn1 + US can provide an effective clinical solution for PD‐1 antibody treatment of advanced HCC.

The advantages of BiO_2‐X_/siFn1 + US treatment are as follows: (I) BiO_2‐X_ has good biosafety and biocompatibility. BiO_2‐X_ is used to deliver siFn1, which can effectively deliver therapeutic drugs to tumors and has good targeting. (II) Using US‐controlled irradiation of the tumor site to minimize systemic toxicity, and US‐triggered BiO_2‐X_ has a good sonothermal effect. The sonothermal effect can kill tumor cells and activate a strong anti‐tumor immune response. At the same time, it can effectively reshape the TME to make ECM loose, enhance the infiltration of drugs and T cells in the tumor site, and enhance the therapeutic effect. Finally, the sonothermal effect can promote the transfection efficiency of siFn1. (III) The design of BiO_2‐X_/siFn1 + US treatment was optimized according to the pattern of tumor PD‐1 antibody resistance, and siFn1 was delivered to reduce FN synthesis. The TME resistant to PD‐1 antibody therapy was successfully reversed. At the same time, the reduction of FN synthesis can also reverse the anoikis resistance of tumor cells, reduce circulating tumor cells and inhibit tumor metastasis.

Although we have used mice to establish a tumor model with closely similar pathogenesis, it remains unclear whether comparable immune alterations also occur in human following BiO_2‐X_/siFn1 + US treatment. As a sonothermal conversion material, BiO_2‐X_ has a good safety prospect. However, the metabolic pathways of nanoformulation still need to be systematically evaluated. Finally, this study focuses on FN and collagen‐I, while ECM components are complex, the role of other components (such as hyaluronic acid) in treatment and whether multi‐target synergistic intervention is needed are worthy of future research.

Our US‐controlled BiO_2‐X_ nanoplatform integrates strategies such as sonothermal therapy, ECM remodeling, and immune activation. By effectively reshaping the ECM components and immune microenvironment of HCC, it significantly enhances drug accumulation and immune cell infiltration, and reverses ECM‐mediated metastasis tendency and immunotherapy resistance, providing a promising new solution to overcome the resistance of advanced HCC immunotherapy. The present study yields novel insights into the dual functions of tumor killing and reversing PD‐1 antibody treatment resistance, constituting a significant step forward in the development of multifunctional materials for cancer treatment.

## Author Contributions

H.T., S.Z., and B.W. devised the overall research strategy and designed the experiments; Y.C., H.S., and H.T. conceived and organized the image layout; W.X. and C.W. conducted some experiments; H.Y. and H.X. wrote the paper; W.C. provided overall supervision throughout the study.

## Conflicts of Interest

The authors declare no conflicts of interest.

## Supporting information




**Supporting File**: advs77337‐sup‐0001‐SuppMat.docx.

## Data Availability

The data that support the findings of this study are available on request from the corresponding author. The data are not publicly available due to privacy or ethical restrictions.

## References

[1] M. Zhou , H. Wang , X. Zeng , et al., “Mortality, Morbidity, and Risk Factors in China and its Provinces, 1990–2017: A Systematic Analysis for the Global Burden of Disease Study 2017,” The Lancet 394, no. 10204 (2019): 1145–1158, 10.1016/s0140-6736(19)30427-1. PMC6891889 31248666

[2] T. F. Greten , C. W. Lai , G. Li , and K. F. Staveley‐O'Carroll , “Targeted and Immune‐Based Therapies for Hepatocellular Carcinoma,” Gastroenterology 156, no. 2 (2019): 510–524, 10.1053/j.gastro.2018.09.051. PMC6340758 30287171

[3] D. M. Xiang , W. Sun , T. Zhou , et al., “Oncofetal Hlf Transactivates C‐Jun to Promote Hepatocellular Carcinoma Development and Sorafenib Resistance,” Gut 68, no. 10 (2019): 1858–1871, 10.1136/gutjnl-2018-317440. 31118247

[4] S. Lamorte , R. Quevedo , R. Jin , et al., “Lymph Node Macrophages Drive Immune Tolerance and Resistance to Cancer Therapy by Induction of the Immune‐Regulatory Cytokine Il‐33,” Cancer Cell 43, no. 5 (2025): 955–969.e10, 10.1016/j.ccell.2025.02.017. PMC12074877 40054466

[5] E. N. Baruch , F. O. Gleber‐Netto , P. Nagarajan , et al., “Cancer‐Induced Nerve Injury Promotes Resistance to Anti‐Pd‐1 Therapy,” Nature 646, no. 8084 (2025): 462–473, 10.1038/s41586-025-09370-8. PMC12406299 40836096

[6] F. Xu , T. Jin , Y. Zhu , and C. Dai , “Immune Checkpoint Therapy in Liver Cancer,” Journal of Experimental & Clinical Cancer Research 37, no. 1 (2018): 110, 10.1186/s13046-018-0777-4. PMC5975687 29843754

[7] E. Triantafyllou , C. L. C. Gudd , and L. A. Possamai , “Immune‐Mediated Liver Injury From Checkpoint Inhibitors: Mechanisms, Clinical Characteristics and Management,” Nature Reviews Gastroenterology & Hepatology 22, no. 2 (2025): 112–126, 10.1038/s41575-024-01019-7. 39663461

[8] A. Bagaev , N. Kotlov , K. Nomie , et al., “Conserved Pan‐Cancer Microenvironment Subtypes Predict Response to Immunotherapy,” Cancer Cell 39, no. 6 (2021): 845–865.e7, 10.1016/j.ccell.2021.04.014. 34019806

[9] B. Wu , B. Zhang , B. Li , H. Wu , and M. Jiang , “Cold and Hot Tumors: From Molecular Mechanisms to Targeted Therapy,” Signal Transduction and Targeted Therapy 9, no. 1 (2024): 274, 10.1038/s41392-024-01979-x. PMC11491057 39420203

[10] S. Wang , X. Yuan , Z. Yang , et al., “Matrix Stiffness‐Dependent Pd‐L2 Deficiency Improves Smyd3/Xct‐Mediated Ferroptosis and the Efficacy of Anti‐Pd‐1 in Hcc,” Journal of Advanced Research 73 (2025): 265–282, 10.1016/j.jare.2024.08.021. PMC12225897 39159723

[11] M. Yu , D. T. Ting , S. L. Stott , et al., “Rna Sequencing of Pancreatic Circulating Tumour Cells Implicates Wnt Signalling in Metastasis,” Nature 487, no. 7408 (2012): 510–513, 10.1038/nature11217. PMC3408856 22763454

[12] L. Wang , C. Li , J. Wang , et al., “Transformable ECM Deprivation System Effectively Suppresses Renal Cell Carcinoma by Reversing Anoikis Resistance and Increasing Chemotherapy Sensitivity (Adv. Mater. 43/2022),” Advanced Materials 34, no. 43 (2022): 2270299, 10.1002/adma.202203518. 36004775

[13] H.‐J. Han , J. Y. Sung , S.‐H. Kim , et al., “Fibronectin Regulates Anoikis Resistance via Cell Aggregate Formation,” Cancer Letters 508 (2021): 59–72, 10.1016/j.canlet.2021.03.011. 33771684

[14] A. Naba , “Mechanisms of Assembly and Remodelling of the Extracellular Matrix,” Nature Reviews Molecular Cell Biology 25, no. 11 (2024): 865–885, 10.1038/s41580-024-00767-3. PMC11931590 39223427

[15] J. Unga and M. Hashida , “Ultrasound Induced Cancer Immunotherapy,” Advanced Drug Delivery Reviews 72 (2014): 144–153, 10.1016/j.addr.2014.03.004. 24680708

[16] L. Yang , B. Tian , Y. Xie , et al., “Oxygen‐Vacancy‐Rich Piezoelectric BiO_2−x_ Nanosheets for Augmented Piezocatalytic, Sonothermal, and Enzymatic Therapies,” Advanced Materials 35, no. 29 (2023): 2300648, 10.1002/adma.202300648. 37058740

[17] Y. Xu , Y. Wang , H. Bi , et al., “A Sonopiezoelectric Z‐Scheme Nanoplatform Synergizing Trace Ag(+) for Eradicating Methicillin‐Resistant Staphylococcus Aureus‐Induced Pneumonia,” ACS Nano 20 (2026): 3593–3609, 10.1021/acsnano.5c16768. 41540854

[18] H. Fu , L. Feng , Q. Liang , and X. Xiao , “Defect‐Rich BiO_2_ _−_ x ‐BP Composite Nanoplatform: A Synergistic Approach for X‐Ray and Near‐Infrared‐Enhanced Cancer Radiodynamic Therapy,” Advanced Healthcare Materials 14, no. 10 (2025): 2404815, 10.1002/adhm.202404815. 39935071

[19] Z. Tu , M. Liu , C. Xu , et al., “Functional 2d Nanoplatforms Alleviate Eosinophilic Chronic Rhinosinusitis by Modulating Eosinophil Extracellular Trap Formation,” Advanced Science 11, no. 19 (2024): 2307800, 10.1002/advs.202307800. PMC11109617 38477549

[20] S. Li , J. Yu , Y. Shen , et al., “Transdermal Microneedle‐Assisted Ultrasound‐Enhanced Crispra System to Enable Sono‐Gene Therapy for Obesity,” Nature Communications 16, no. 1 (2025): 1499, 10.1038/s41467-025-56755-4. PMC11811159 39929846

[21] D. Song , Q. Liu , Z. Yan , et al., “WSGC@FA@PEG/PEI‐SPIONs Mitigate Chemoresistance in Gastric Adenocarcinoma by Modulating the Notch Signaling Pathway and Mitophagy,” Advanced Science 12, no. 36 (2025): 15840, 10.1002/advs.202415840. PMC12463016 40830080

[22] M. Guo , X. Zhang , J. Liu , et al., “Few‐Layer Bismuthene for Checkpoint Knockdown Enhanced Cancer Immunotherapy With Rapid Clearance and Sequentially Triggered One‐for‐All Strategy,” ACS Nano 14, no. 11 (2020): 15700–15713, 10.1021/acsnano.0c06656. 33155807

[23] X. Liu , N. Rong , Z. Tian , et al., “Acoustothermal Transfection for Cell Therapy,” Science Advances 10, no. 16 (2024): adk1855, 10.1126/sciadv.adk1855. PMC11023511 38630814

[24] T. Tan , H. Hu , H. Wang , et al., “Bioinspired Lipoproteins‐Mediated Photothermia Remodels Tumor Stroma to Improve Cancer Cell Accessibility of Second Nanoparticles,” Nature Communications 10, no. 1 (2019): 3322, 10.1038/s41467-019-11235-4. PMC6658501 31346166

[25] Y. Zhang , X. Dong , Y. Zhang , et al., “Biomaterials to Regulate Tumor Extracellular Matrix in Immunotherapy,” Journal of Controlled Release 376 (2024): 149–166, 10.1016/j.jconrel.2024.10.010. 39389365

[26] Q. Tang and A. Khvorova , “Rnai‐Based Drug Design: Considerations and Future Directions,” Nature Reviews Drug Discovery 23, no. 5 (2024): 341–364, 10.1038/s41573-024-00912-9. PMC11144061 38570694

[27] K. A. Whitehead , R. Langer , and D. G. Anderson , “Knocking Down Barriers: Advances in Sirna Delivery,” Nature Reviews Drug Discovery 8, no. 2 (2009): 129–138, 10.1038/nrd2742. PMC7097568 19180106

[28] Z. Xiang , J. Li , Y. Xu , et al., “Cell Differentiation‐Related Signaling Pathways in Hepatocellular Carcinoma Metastasis,” Cancer Letters 627 (2025): 217846, 10.1016/j.canlet.2025.217846. 40456400

[29] L. Chen , L. Diao , Y. Yang , et al., “Cd38‐Mediated Immunosuppression as a Mechanism of Tumor Cell Escape From Pd‐1/Pd‐L1 Blockade,” Cancer Discovery 8 (2018): 1156–1175, 10.1158/2159-8290.Cd-17-1033. PMC6205194 30012853

[30] Z. Jiang , T. Li , H. Cheng , et al., “Nanomedicine Potentiates Mild Photothermal Therapy for Tumor Ablation,” Asian Journal of Pharmaceutical Sciences 16 (2021): 738–761, 10.1016/j.ajps.2021.10.001. PMC8739255 35027951

[31] G. Sahay , W. Querbes , C. Alabi , et al., “Efficiency of Sirna Delivery by Lipid Nanoparticles is Limited by Endocytic Recycling,” Nature Biotechnology 31, no. 7 (2013): 653–658, 10.1038/nbt.2614. PMC3814166 23792629

[32] J. Gilleron , W. Querbes , A. Zeigerer , et al., “Image‐Based Analysis of Lipid Nanoparticle–Mediated siRNA Delivery, Intracellular Trafficking and Endosomal Escape,” Nature Biotechnology 31, no. 7 (2013): 638–646, 10.1038/nbt.2612. 23792630

[33] Y. Chen , Y. Huang , Q. Li , et al., “Targeting Xkr8 via Nanoparticle‐Mediated In Situ Co‐Delivery of Sirna and Chemotherapy Drugs for Cancer Immunochemotherapy,” Nature Nanotechnology 18, no. 2 (2023): 193–204, 10.1038/s41565-022-01266-2. PMC9974593 36424448

[34] J. Liu and F. Shao , ““Boosting” Tumor Immunity in Elderly Mice by Hyperactivating Dendritic Cells,” Cell 187, no. 15 (2024): 3885–3887, 10.1016/j.cell.2024.06.025. 39059365

[35] P. Rodríguez‐Silvestre , M. Laub , P. A. Krawczyk , et al., “Perforin‐2 is a Pore‐Forming Effector of Endocytic Escape in Cross‐Presenting Dendritic Cells,” Science 380, no. 6651 (2023): 1258–1265, 10.1126/science.adg8802. PMC7614779 37347855

[36] Z. Yang , D. Gao , J. Zhao , et al., “Thermal Immuno‐Nanomedicine in Cancer,” Nature Reviews Clinical Oncology 20, no. 2 (2023): 116–134, 10.1038/s41571-022-00717-y. 36604531

[37] H.‐R. Kim , J.‐S. Park , J.‐H. Park , et al., “Cell‐Permeable Transgelin‐2 as a Potent Therapeutic for Dendritic Cell‐Based Cancer Immunotherapy,” Journal of Hematology & Oncology 14, no. 1 (2021): 43, 10.1186/s13045-021-01058-6. PMC7968273 33731208

[38] Z. Xishan , A. Guangyu , S. Yuguang , and Z. Hongmei , “The Research on the Immuno‐Modulatory Defect of Mesenchymal Stem Cell From Chronic Myeloid Leukemia Patients,” Journal of Experimental & Clinical Cancer Research 30, no. 1 (2011): 47, 10.1186/1756-9966-30-47. PMC3095541 21535879

[39] Y. Wang , S. Cheng , J. S. Fleishman , et al., “Targeting Anoikis Resistance as a Strategy for Cancer Therapy,” Drug Resistance Updates 75 (2024): 101099, 10.1016/j.drup.2024.101099. 38850692

[40] P. Shaw , A. Dey Bhowmik , M. S. Gopinatha Pillai , N. Robbins , S. K. D. Dwivedi , and G. Rao , “Anoikis Resistance in Cancer: Mechanisms, Therapeutic Strategies, Potential Targets, and Models for Enhanced Understanding,” Cancer Letters 624 (2025): 217750, 10.1016/j.canlet.2025.217750. PMC13014371 40294841

[41] Z. Xu , D. Yang , T. Long , et al., “Ph‐Sensitive Nanoparticles Based on Amphiphilic Imidazole/Cholesterol Modified Hydroxyethyl Starch for Tumor Chemotherapy,” Carbohydrate Polymers 277 (2022): 118827, 10.1016/j.carbpol.2021.118827. 34893244

[42] J. Yu , B. Zhou , S. Zhang , et al., “Design of a Self‐Driven Probiotic‐Crispr/Cas9 Nanosystem for Sono‐Immunometabolic Cancer Therapy,” Nature Communications 13, no. 1 (2022): 7903, 10.1038/s41467-022-35580-z. PMC9780327 36550159

[43] Y. Dong , Q. Zheng , Z. Wang , et al., “Higher Matrix Stiffness as an Independent Initiator Triggers Epithelial‐Mesenchymal Transition and Facilitates Hcc Metastasis,” Journal of Hematology & Oncology 12, no. 1 (2019): 112, 10.1186/s13045-019-0795-5. PMC6839087 31703598

[44] M. Li , H. He , X. Zhao , et al., “Burden of Liver Cancer and Underlying Etiologies in China From 1990 to 2021: A Systematic Analysis From the Global Burden of Disease Study 2021,” Chinese Medical Journal 139, no. 3 (2026): 415–423, 10.1097/cm9.0000000000003828. PMC12875693 41481853

[45] S. Dai , Y. Chen , W. Cai , et al., “Combination Immunotherapy in Hepatocellular Carcinoma: Synergies Among Immune Checkpoints, Tkis, and Chemotherapy,” Journal of Hematology & Oncology 18, no. 1 (2025): 85, 10.1186/s13045-025-01739-6. PMC12465164 41013723

[46] C. Meng , H. Zhang , X. Yi , et al., “Reversal of Tumour Immune Evasion via Enhanced Mhc‐Class‐I Antigen Presentation by a Dual‐Functional Rna Regulated System,” Molecular Cancer 24, no. 1 (2025): 258, 10.1186/s12943-025-02480-x. PMC12522306 41088223

[47] C. Yu , D. Chen , D. Zhu , et al., “Dual Strategy of Ca^2+^ Influx and Collagen Denaturation to Remodel the Extracellular Matrix and Amplify Sonopiezoelectric Therapy,” Advanced Materials 37, no. 33 (2025): 2501642, 10.1002/adma.202501642. 40444340

[48] P. Xie , M. Yu , B. Zhang , et al., “Crkl Dictates Anti‐Pd‐1 Resistance by Mediating Tumor‐Associated Neutrophil Infiltration in Hepatocellular Carcinoma,” Journal of Hepatology 81, no. 1 (2024): 93–107, 10.1016/j.jhep.2024.02.009. 38403027

[49] X. Song , Q. Zhang , M. Chang , et al., “Nanomedicine‐Enabled Sonomechanical, Sonopiezoelectric, Sonodynamic, and Sonothermal Therapy,” Advanced Materials 35, no. 31 (2023): 2212259, 10.1002/adma.202212259. 36812400

[50] J. Li , Y. Luo , Z. Zeng , et al., “Precision Cancer Sono‐Immunotherapy Using Deep‐Tissue Activatable Semiconducting Polymer Immunomodulatory Nanoparticles,” Nature Communications 13, no. 1 (2022): 4032, 10.1038/s41467-022-31551-6. PMC9276830 35821238

[51] S. Sun , X. Huang , N. Yang , et al., “Fluorinated Titanium Oxide (Tio_2‐X_F_X_) Nanospindles as Ultrasound‐Triggered Pyroptosis Inducers to Boost Sonodynamic Immunotherapy,” ACS Nano 18 (2024): 19756–19770, 10.1021/acsnano.4c05448. 39010657

