# Supplementary material for: Defect‐Engineered BiO2‐X Nanosheets Mediate Sono‐Thermomechanical ECM Remodeling for Hepatocellular Carcinoma Immunotherapy
Source: Adv Sci (Weinh). 2026 Aug 29;13(63):e77337. doi: 10.1002/advs.77337 (PMC13525413; doi:10.1002/advs.77337)
Supplement: Supplementary file 1 [file ADVS-13-e77337-s001.docx]

Supporting Information for

Defect-Engineered BiO_2-X_ Nanosheets Mediate Sono-Thermomechanical ECM Remodeling for Hepatocellular Carcinoma Immunotherapy

Authors

*Huimin Tian^1^****^#^****, Shen Zhang ^2, 3^****^#^****, Bolin Wu^1^****^#^****, Yichi Chen^1^, Haitao Shang^1^, Haoyan Tan^1^, Chunyue Wang^1^, Weichen Xu^3^, Huixiong Xu^2^*, Haohao Yin^2^*, Wen Cheng^1^**

Affiliations

H. Tian, B. Wu, Y. Chen, H. Shang, H. Tan, C. Wang, W. Cheng

^1^Department of Ultrasound, Harbin Medical University Cancer Hospital, Harbin, Heilongjiang 150081, P. R. China.

E-mail: chengwen@hrbmu.edu.cn

S. Zhang, H. Yin, H. Xu

^2^Department of Ultrasound, Institute of Ultrasound in Medicine and Engineering, Zhongshan Hospital, Fudan University, Shanghai, 200032, P. R. China.

E-mail: [yin.haohao@zs-hospital.sh.cn](mailto:yin.haohao@zs-hospital.sh.cn); [xuhuixiong@126.com](mailto:xuhuixiong@126.com)

S. Zhang, W. Xu

^3^Department of Medical Ultrasound, Center of Minimally Invasive Treatment for Tumor, Shanghai Tenth People’s Hospital, School of Medicine, Tongji University, Shanghai 200072, P. R. China.

**^#^**These authors contributed equally: Huimin Tian, Shen Zhang, Bolin Wu

**Materials and methods**

**Ethical approval**

All animal procedures were conducted in accordance with protocols approved by the Institutional Animal Care and Use Committee of Shanghai Tenth People’s Hospital (protocol no. SHDSYY-2024-Y3325-2). Animal husbandry and experimental procedures complied with the regulations of the National Ministry of Health regarding animal research. Female mice were used in all animal experiments because of their relatively docile temperament, and sex was considered unlikely to influence the experimental outcomes. A maximum tumor burden threshold of 1500 mm^3^ was established for the mice, and this limit was not exceeded in any of the animal experiments.

**Transcriptome sequencing**

Hepa1-6 tumor cells (1 × 10^6^) were injected subcutaneously into the inguinal area of 6-week-old female C57BL/6 mice to establish a subcutaneous tumor model. Mice were randomly assigned to two groups (*n* = 15) according to the mean tumor volume, including anti-PD-1 groups and the IgG isotype control group. For antibody-based drug intervention, an anti-PD-1 antibody (RMP1-14; BioXCell) or rat immunoglobulin G (BioXCell, BE0089) (100 μg per mouse) was injected intraperitoneally every 3 days after tumor implantation. At day 30, tumor tissues resistant to anti-PD-1 treatment and tumor tissues sensitive to anti-PD-1 treatment were collected, followed by nucleic acid extraction and full transcriptome sequencing.

**Preparation of BiO_2-X_ NSs**

BiO_2-X_ NPs were synthesized via a hydrothermal approach. First, 1.40 g of NaBiO_3_ were dispersed in and 2.4 mol L^−1^ NaOH solution (30 mL). The obtained solution was stirred for 2 h at ambient temperature, followed by transfer of the suspension to a high-pressure reactor. The autoclave was then transferred to an oven for hydrothermal treatment at 180℃ for 5 h. Thereafter, the centrifugally collected BiO_2−X_ NPs precipitates were rinsed five times with distilled water and dried under vacuum at 60℃ for 8 h. To obtain thinner BiO_2-X_ NSs, ultrasonic homogenizer was used to exfoliate BiO_2-X_ NPs. The exfoliation conditions were as follows: The BiO_2-X_ NPs were suspended in a deionized water/ethanol mixture and subjected to ultrasonication for 8 h to achieve size reduction. The obtained solution was centrifuged, the supernatant was collected, and sterile water containing 50% ethanol was added. After mixing evenly, the solution was ultrasonically treated at a frequency of 40 kHz for 12 h. The obtained sample was centrifuged and the supernatant was discarded. The resulting precipitate was BiO_2-X_ NSs and washed with sterile water for 5 times and finally dispersed in sterile water.

**Synthesis of BiO_2−X_/siFn1**

Briefly, PEI and mPEG-CHO were first mixed at a weight ratio of 2:1 under pH 7.4 conditions for 30 min to obtain mPEG-d-PEI. Subsequently, BiO_2-X_ NSs were added to the mPEG-d-PEI solution to prepare mPEG-d-PEI-coated BiO_2-X_ (BiO_2-X_@PP). The siRNA loading capacity of the nanosystem was then evaluated by incubating BiO_2-X_@PP with siRNA at different BiO_2-X_/siRNA weight ratios. After 30 min of incubation, 5× loading buffer was added, and the samples were analyzed by 2% agarose gel electrophoresis containing Gel Red at 80 V for 20 min.

**Characterization**

Transmission electron microscopy (TEM) (FEI Talos F200X) was performed to observe the morphology and corresponding elements mapping of the delivery nanosystem. Atomic force microscopy (AFM) was detected by Bruker Multimode 8-HR. The dynamic light scattering (DLS) and zeta potential measurements were measured on a Nano ZS 960 Malvern Zetasizer Nanoseries (Malvern Instrument Ltd. UK). The phase and crystallinity of the as-synthesized materials were determined by X-ray diffraction (XRD) on a Bruker D8 Advance diffractometer equipped with Cu Kα radiation (λ = 1.5406 Å). The electron paramagnetic resonance (EPR) experiments were conducted at room temperature using an EPR spectrometer (Bruker E500, Bruker, Germany). The UV–vis spectrum was collected on UV-1900i Shimadzu spectroscope. The RNase stability of BiO_2−X_/siFn1 was measured by agarose gel electrophoresis. The confocal laser scanning microscope (ZEISS LSM900) was used to acquire the confocal images. The flow cytometry of cells was detected by using flow cytometry (BD LSRFortessa).

**Sonothermal performance measurements**

To investigate the effect of BiO_2−X_ concentration on sonothermal performance, BiO_2−X_ NS dispersions at different concentrations (0, 10, 20, 50, 100, and 200 μg mL^−1^) were exposed to continuous ultrasound (US) irradiation (1.0 W cm^−2^), and the temperature changes were monitored using an infrared thermal camera. Deionized water was irradiated with US at different power densities (0.6, 0.8, 1.0 W cm^−2^) for 3 min, and the temperature profiles of each group were recorded. Subsequently, the temperature profiles of BiO_2−X_ NS dispersions (50 μg mL^−1^) under US irradiation at different power densities (0.4, 0.6, 0.8, 1.0, 1.2 and 1.4 W cm^−2^) for 3 min were recorded. In addition, the stability of the sonothermal properties of BiO_2−X_ NSs was evaluated by monitoring the heating and cooling behavior of an aqueous BiO_2−X_ NS dispersion (200 μg mL^−1^) during three cycles of US irradiation for 150 s, followed by natural cooling to 26 ℃ after cessation of US exposure.

**Cell culture**

The NIH-3T3, Hepa1-6, and Hepa1-6-luciferase (Hepa1-6-Luc) tumor cells were cultured in Dulbecco’s Modified Eagle Medium (DMEM, Gibco) supplemented with 10% fetal bovine serum (FBS, Gibco) and 1% penicillin-streptomycin. H22-Luc tumor cells were cultured in RPMI 1640 medium (Gibco) containing 10% FBS and 1% penicillin-streptomycin. All cells were cultured at 37℃ in a humidity atmosphere containing 5% CO_2_. The anoikis-resistance model was established by continuously culturing Hepa1-6 cells in ultra-low-attachment 6-well plates (Corning, NY, USA) for 7 days, after which they were transferred to standard plates to allow adherence for 24 h. Re-adherent cells were deemed anoikis-resistance Hepa1-6 cells.

**Construction of stable Fn1-knockdown cell lines**

Hepa1-6 tumor cells were transduced with shRNA targeting Fn1 (encoding fibronectin, FN) to establish FN-knockdown cell lines following the manufacturer’s instructions. Meanwhile, cells transduced with non-targeting vector were generated in parallel as shControl group. Subsequently, cells were subjected to two rounds of puromycin selection at a concentration of 10 μg mL^−1^, with each selection period lasting 48–72 h in accordance with the manufacturer’s protocol. The efficiency of gene knockdown was finally validated via western blotting analysis.

**Analysis of cellular uptake and lysosomal escape of BiO_2−X_/siFn1**

Hepa1-6 cells were seeded into confocal dishes and cultured for 24 h. To analyze the lysosomal escape of the BiO_2−X_/siFn1 nanosheets, siFn1 was labeled with Cy5.5-BiO_2−X_ to monitor its intracellular distribution. After incubated with Cy5.5-labeled nanosheets for 4 h, the cells were exposed to US irradiation (1.0 MHz, 1.0 W cm^−2^, 50% duty cycle, 3 min). The cell nuclei and lysosomes were stained by DAPI and LysoTracker Green (Beyotime), respectively, and the intracellular distribution of BiO_2−X_/siFn1 was monitored by confocal laser scanning microscopy (CLSM) observation.

**Evaluation of cell viability**

Cell viability was evaluated using a Cell Counting Kit-8 (CCK-8) assay and a Calcein-AM/PI staining assay. Briefly, Hepa1-6 cells were seeded in 96-well plates (10^4^ per well) and then cultured overnight at 37℃ in an incubator. After that, treatments were conducted, including (1) incubation with different concentrations of BiO_2−X_ (0, 25, 50, 100, 200, and 400 μg mL^−1^), (2) incubation with following treatments: phosphate-buffered saline (PBS), US only (1.0 MHz, 1.0 W cm^−2^, 50% duty cycle, 3 min), BiO_2−X_ (50 μg mL^−1^), siFn1 (1.33 μg mL^−1^), BiO_2−X_/siFn1, BiO_2−X_ (50 μg mL^−1^) + US (1.0 MHz, 1.0 W cm^−2^, 50% duty cycle, 3 min), BiO_2−X_/siFn1 + US (1.0 MHz, 1.0 W cm^−2^, 50% duty cycle, 3 min). The cells were further incubated for another 24 h and washed three times with PBS. Then, 100 μL of CCK-8 reagent (Beyotime Biotechnology) was introduced into each well and incubated for a further 1 h, after which the absorbance at 450 nm was measured with a microplate reader (Bio-Tek ELx800, USA) to determine cell viability.

For Calcein-AM/PI staining assay, Hepa1-6 cells were seeded into 6-well plates and incubated for 24 h. After receiving different treatments, including (G1) PBS, (G2) US irradiation, (G3) BiO_2−X_, (G4) siFn1, (G5) BiO_2−X_/siFn1, (G6) BiO_2−X_ + US, and (G7) BiO_2−X_/siFn1 + US, the cells were stained with Calcein-AM/PI solution for 30 min, followed by CLSM observation.

**Quantitative reverse transcription polymerase chain reaction (qRT-PCR)**

RNA expression in vitro was detected using qRT-PCR. Total RNA was extracted using the Eastep total RNA extraction kit (Promega, Madison, WI, USA), following standard procedures. The isolated total RNA was subsequently reverse-transcribed into cDNA using the HiScript®II1st strand cDNA synthesis kit. Quantitative real-time PCR was conducted using SYBR Green PCR master mix, with GAPDH serving as the internal reference for normalization. All primers used in this study were purchased from Sangon Biotech (Shanghai, China).

**Preparation of bone marrow-derived dendritic cells (BMDCs)**

BMDCs were isolated from the femurs and tibias of female C57BL/6 mice aged 6-8 weeks. Briefly, following euthanasia, the femurs and tibias from the hind limbs were harvested, and bone marrow cells were gently flushed out with precooled PBS. After centrifugation, the cells were resuspended in RPMI 1640 medium supplemented with 2 mM l -glutamine, 10% FBS, granulocyte-macrophage colony-stimulating factor (GM-CSF, 20 ng mL^−1^), IL-4 (10 ng mL^−1^), penicillin (50 U mL^−1^), and streptomycin (50 ng mL^−1^), and then seeded into sterile plastic Petri dishes (10 mL per dish) at a density of 5 × 10^6^ cells per dish. The culture medium was replaced on day 3, and BMDCs were harvested on day 7 for subsequent experiments.

**Western blot analysis**

Hepa1-6 cells were maintained in complete medium and subjected to the indicated treatments. Cells were then lysed in RIPA buffer containing a protease inhibitor cocktail. Protein concentrations were determined using the Pierce BCA Protein Assay Kit, and samples were denatured in Laemmli buffer supplemented with 150 mM DTT at 95℃ for 5 min. Subsequently, equal amounts of protein (30 μg) were resolved by 4-15% SDS-PAGE (Bio-Rad) and transferred to a 0.45 μm polyvinylidene difluoride (PVDF) membrane. Following blocking with 5% bovine serum albumin (BSA), the membrane was incubated with polyclonal rabbit anti-FN antibody (1:1000) overnight at 4℃. After incubation with horseradish peroxidase (HRP)-linked secondary antibody (1:5000 dilution 1 h at room temperature), Western Lightning Chemiluminescence Reagent Plus (PerkinElmer) was used for visualization. Images were acquired with the ChemiDoc Touch Imaging System (Bio-Rad) and analyzed with Image Lab software (Bio-Rad).

**Immunofluorescence**

In this assay, seven groups were set. Hepa1-6 cells were seeded in confocal dish (1 × 10^4^ cells/well) for 12 h. In the BiO_2−X_/siFn1 + US group, the cells were first treated with BiO_2−X_/siFn1 for 4 h. After treatment, the cells were subjected to US irradiation (1.0 MHz, 1.0 W cm^−2^, 50% duty cycle) for 3 min and then incubated for a further 20 h. The cells were subsequently washed with PBS, fixed in 4% paraformaldehyde for 20 min, and incubated with anti-collagen-I antibody for 4 h at room temperature. Afterward, Alexa Fluor®555-conjugated secondary antibody was used for CLSM observation, and Hepa1-6 cell nuclei were stained with DAPI.

**In vitro assessment of gene silencing by siRNA-loaded BiO_2-X_**

Hepa1-6-Luc cells were plated in 12-well plates at a density of 10^6^ cells per well and maintained in 1 mL of DMEM containing 10% FBS for 24 h. Thereafter, the cells were treated with siLuc-loaded NSs for an additional 24 h, washed and replenished with fresh medium, and then incubated with D-luciferin for 5 min. Bioluminescence was measured using an In Vivo Imaging System to determine firefly Luc expression. Average radiance (p s^−1^ cm^−2^ sr^−1^) in the selected regions of interest was quantified with AniView software, and GraphPad software was used to generate plots of radiance changes.

**In vivo gene knockdown**

Hepa1-6-Luc cells were subcutaneous (s.c.) inoculated into the right lower abdomen of C57BL/6 mice. SiLuc or control siRNA (siCT)-loaded BiO_2-X_ NSs were injected into Hepa1-6-Luc tumor bearing mice at a dose of 133.3 μg siRNA kg^−1^. Gene knockdown efficiency was assessed on three occasions by whole-body bioluminescence imaging, performed one day after the first, second, and third injections, which corresponded to days 10, 15, and 20 post-tumor inoculation, respectively. Mice were anesthetized for the first two imaging and euthanized for the final imaging.

**In vitro cell internalization**

Hepa1-6 cells were seeded on the confocal dish at a density of 5 × 10^4^ cells per well and incubated overnight. Then cells were washed with PBS twice and fixed with 4% formaldehyde for 20 min. And then cell cytoskeleton and nucleus were stained with Alexa Fluor 488 phalloidin (Beyotime) and DAPI respectively with standard procedures of manufacturers.

**Reactive oxygen species (ROS) measurement**

The 1,3-diphenylisobenzofuran (DPBF; MedChemExpress) was employed to quantitatively evaluate the generation of ^1^O_2_ by the samples. BiO_2−X_/siFn1 and BiO_2−X_ was dispersed in 2 mL of PBS solution and mixed with 2 mL of ethanol-containing DPBF solution (50 μm). After homogenization in the dark, the resultant mixture was subjected to ultrasonic irradiation (1 MHz, 1 W cm^−2^, 50% duty cycle) for 0, 1, 2, and 3 min, respectively. Control groups were set up, including the DPBF + US group. Subsequently, the UV-visible absorption spectrum of the DPBF solution was recorded from 300 to 500 nm using a UV–vis spectrophotometer (UV1800PC, AoE, China).

**In vitro immunogenic cell death expression assay**

Hepa1-6 cells were seeded in six-well plates at a density of 1 × 10^5^ cells per well and cultured overnight to allow sufficient cell attachment. The cells were then treated according to the indicated experimental groups and further incubated for 12 h. The culture supernatants from each group were collected and centrifuged to remove cell debris. High mobility group box 1 (HMGB1) release in the supernatants was then measured using an enzyme-linked immunosorbent assay (ELISA) kit according to the manufacturer’s instructions.

For the detection of cell-surface calreticulin (CRT), the treated cells were collected and washed three times with pre-cooled PBS. To specifically detect CRT exposure on the cell membrane, the cells were stained without permeabilization. The cells were then incubated with a rabbit monoclonal anti-CRT antibody for 1 h. After washing with PBS, the cells were incubated with goat anti-rabbit IgG H&L (CoraLite® Plus 488) secondary antibody (Proteintech, Cat. No. RGAR002) for 1 h in the dark. After another three washes with PBS, the cells were resuspended in PBS and analyzed by flow cytometry to detect the surface expression of CRT on Hepa1-6 cells. Unstained controls and secondary antibody-only controls were included for gating and to exclude nonspecific fluorescence signals.

**Anoikis detection**

To evaluate anoikis, anoikis-resistant Hepa1-6 cells were maintained in ultra-low-attachment plates for 24 h after different treatments. Anoikis was detected using an Annexin V-FITC/PI Apoptosis Detection Kit (BD Biosciences, Franklin Lakes, NJ, USA). In brief, 5 × 10^5^ cells were harvested, incubated with Annexin V-FITC/PI for 15 min in the dark at room temperature, and then subjected to flow cytometric analysis (Beckman Coulter, Indianapolis, IN, USA) to determine the anoikis.

**Transwell assay**

The cells treated with PBS, US, BiO_2−X_, siFn1, BiO_2−X_/siFn1, BiO_2−X_ + US, and BiO_2−X_/siFn1 + US were seeded in a transwell chamber (Corning) with 8.0 μm pores, and the chamber was placed in a 24-well plate. For the assessment of cell migration, 5 × 10^4^ cells were plated in the upper chamber in 100 μL of serum-free DMEM, whereas 800 μL of DMEM containing 10% FBS was placed in the lower chamber as a chemoattractant. The upper chamber was precoated with Matrigel, and an analogous protocol was adopted for the invasion assay. Following 24 h of incubation, cells in the lower chamber were fixed with 4% paraformaldehyde, stained with 0.1% crystal violet, and quantified in three fields per well.

**Hemolysis assay**

Whole blood was obtained through centrifugation and washing to acquire red blood cells (RBCs), and a 5% red blood cell suspension was prepared. After 1 mL of the suspended red blood cells were incubated with different concentrations (0, 10, 20, 50 and 100 μg mL^−1^) of BiO_2−X_/siFn1 suspension for 1h or 4h at 150 rpm in a shaker at 37℃, the red blood cell suspension was aspirated and then centrifuged at 1500 rpm for 10 min. Absorbance values were measured at 540 nm. PBS and 0.1% Triton X-100 were respectively employed as negative and positive controls.

**In vivo safety evaluation**

To assess the biosafety of BiO_2−X_/siFn1, blood samples were obtained for both biochemical and hematological analyses. Specifically, 200 μL of blood was placed in a procoagulant tube containing separation gel and centrifuged for 10 min, and the resulting supernatant was used to evaluate liver and kidney function indices, including AST, ALT, and BUN. In parallel, 100 μL of blood was collected into an anticoagulant tube for routine blood examination, including measurements of RBC, WBC, PLT, and MCV. In addition, major organs, including the heart, liver, spleen, lungs, and kidneys, were harvested, weighed, fixed in 4% paraformaldehyde, processed by dehydration and embedding, and sectioned for HE staining and histopathological observation using light microscopy.

**In vivo biodistribution of BiO_2−X_/siFn1 particles**

BiO_2−X_/siFn1 and siFn1 were incubated with Cy5.5 at room temperature for 2 h. Free Cy5.5 was removed by ultracentrifugation. After tail vein injection of different preparations (PBS, Cy5.5, Cy5.5-siFn1 and BiO_2−X_/siFn1), the mice were imaged at different time points (2 h, 6 h, 12 h, 24 h, 36 h, and 48 h) in vivo to observe the fluorescence intensity of Cy5.5 in the main organs. Twenty-four hours later, the mice were sacrificed, and the principal organs, including the heart, liver, spleen, lungs, kidneys, and tumors, were collected for ex vivo fluorescence imaging.

**Inductively coupled plasma mass spectrometry (ICP-MS)**

BiO_2−X_/siFn1 was intravenously injected into C57BL/6 mice aged 8–10 weeks through the lateral tail vein at a BiO_2−X_ dose of 5 mg kg^−1^. The day of injection was defined as day 0. For the single-injection group, mice were euthanized at 1, 3, 7, and 30 days after injection for sample collection. Urine and feces samples were collected before euthanasia. After euthanasia, five major organs, including the liver, kidney, lung, spleen, and heart, were surgically harvested. Fresh organs and excreta were weighed and digested with HNO₃. The Bi content in the digested samples was quantified using a NexION 300D ICP-MS instrument (PerkinElmer, USA). The Bi levels in organs and feces were expressed as the detected Bi mass divided by the total sample mass (μg Bi g⁻¹), whereas the Bi levels in urine were expressed as the detected Bi mass divided by the total sample volume (μg Bi L⁻¹).

**In vivo antitumor efficiency**

Hepa1-6 tumor cells (1 × 10^6^) were injected subcutaneously into the inguinal area of 6-week-old female C57BL/6 mice to establish a subcutaneous tumor model. These mice were divided at random into 7 groups (*n* = 10): control (200 μL, PBS), US (1.0 MHz, 1.0 W cm^−2^, 50% duty cycle, 3 min), BiO_2-X_ (200 μL, 5 mg kg^−1^), siFn1 (200 μL, 133.3 μg kg^−1^), BiO_2-X_/siFn1 (200 μL, BiO_2-X_ 5 mg kg^−1^, siFn1 133.3 μg kg^−1^), BiO_2-X_ + US (200 μL, 5 mg kg^−1^, US 1.0 MHz, 1.0 W cm^−2^, 50% duty cycle, 3 min), BiO_2-X_/siFn1 + US (200 μL, BiO_2-X_ 5 mg kg^−1^, siFn1 133.3 μg kg^−1^, US 1.0 MHz, 1.0 W cm^−2^, 50% duty cycle, 3 min). Mice received the indicated treatments on days 7, 9, 11, and 13 following tumor inoculation, whereas animals in the US-treated groups were additionally exposed to US irradiation on days 8, 10, 12, and 14. Tumor volume and body weight were measured every 2 days between days 5 and 21. Tumor volume was determined using the formula: (tumor length) × (tumor width)^2^/2. According to humane endpoint criteria, mice were euthanized when they were moribund or when tumor diameter exceeded 2 cm in any direction, provided that tumor burden did not surpass 1500 mm^3^. Survival was monitored from the day of Hepa1-6 cell implantation to day 60. At the end of the treatment period, mice (*n* = 5) were sacrificed, and tumors were excised, fixed in 10% formalin, sectioned, and stained for collagen-I and FN for histopathological analysis.

Subcutaneously inject 1 × 10^6^ Hepa1-6 tumor cells into the inguinal region of 6-week-old female C57BL/6 mice to establish a subcutaneous tumor model. Mice are randomly divided into four groups (*n* = 5): Control group (200 μL PBS), US group (1.0 MHz, 1.0 W cm^−2^, 50% duty cycle, 3 min), siFn1 group (200 μL, 133.3 μg kg^−1^), and US + siFn1 combination group (200 μL, 133.3 μg kg^−1^, US 1.0 MHz, 1.0 W cm^−2^, 50% duty cycle, 3 min). Corresponding treatments are administered to mice on days 7, 9, 11 and 13 post-tumor inoculation; mice receiving US undergo US irradiation on days 8, 10, 12 and 14. Tumor volumes are measured and recorded every two days from day 5 to day 21 post-inoculation for tumor size quantification.

**Cytokine measurement**

Serum was obtained from mice after the indicated treatments, and the concentrations of different cytokines were quantified using ELISA kits according to the manufacturer’s protocols.

**AFM measurement of tumor tissue stiffness**

After the mice were euthanized, the tumor tissues were immediately excised and gently rinsed with pre-cooled PBS to remove residual blood from the tissue surface. The tumors were trimmed to an appropriate size, embedded in optimal cutting temperature (OCT) compound, and rapidly frozen. Serial cryosections with a thickness of 20 μm were then prepared using a cryostat. The sections were mounted on glass slides suitable for AFM and equilibrated in PBS before measurement to maintain tissue hydration. The mechanical properties of the tumor tissues were measured using a NanoWizard V BioAFM system. A spherical indenter probe (E-φ5 μm) with a sphere diameter of 5 μm was used. Before measurement, the deflection sensitivity and spring constant of the probe were calibrated. All samples were measured in a PBS liquid environment. The probe approached the tissue surface vertically, and force–distance curves were acquired from predefined tissue regions. For each region, the scanning area was set to 20 × 20 μm with a spatial resolution of 64 × 64 pixels. The probe approach speed was set to 200 μm s^−1^, and the Z-travel distance for each force curve was set to 3.0 μm. The acquired force–distance curves were processed using the instrument-associated JPK Data Processing software. The apparent Young’s modulus of the tumor tissues was calculated by fitting the curves with a Hertz contact model for a spherical indenter, with the Poisson’s ratio set to 0.5.

**Immunohistochemistry and Immunofluorescence**

Tumors were collected from the mice, embedded in OCT compound, and snap-frozen. The frozen tissues were sectioned using a cryotome and mounted on slides. Immunostaining was then performed with anti-FN primary antibody according to the manufacturer’s protocol, followed by analysis under a confocal microscope.

Tumor tissue was harvested from mice after different treatments. Tumor sections were cut with a frozen section machine, mounted on slides, and labeled with different primary antibodies after embedding and fixation: CRT, HMGB1, Collagen-Ⅰ, CD3, CD4, CD8 and the corresponding secondary antibodies were added according to the manufacturer’s instructions. The nuclei of the tumor cells were stained by DAPI.

**Flow cytometry analysis**

Single-cell suspensions obtained from the tumor, spleen of mice were prepared by gentle mashing and passing through a 70 µm mesh cell strainer. Subsequently, the cells were incubated with Fc receptor-blocking reagent (BD Biosciences) for 15 min at 4℃ and then stained with the following fluorochrome conjugated antibodies staining protocol:

CD45-PerCP, CD11c-FITC, CD80-APC, CD86-PE for analyzing maturation of DCs;

CD45-PerCP, CD3-FITC, CD8-APC, CD4-PE-Cy7 for analyzing proliferation of CD8^+^/CD4^+^ T cells;

CD3-FITC, CD4-PE-Cy7, CD8-APC, CD44-PE, and CD62L-PerCP for analyzing activation of immune memory cells.

CD45-PerCP, CD3-FITC, CD4-PE-Cy7, and FOXP3-PE for analyzing immunosuppressive Treg cells.

Following 30 min of staining, the single-cell suspensions were washed twice with PBS supplemented with 2% FBS and then incubated with PI. Thereafter, the phenotypic profiles of different immune cell subsets were determined by flow cytometry. Data analysis was performed using FlowJo software (version 10). All antibodies were purchased from BioLegend, eBioscience, and R&D Systems.

**The immune response against distant tumor**

Hepa1-6 cells (1 × 10^6^ cells) were first injected into the right inguinal region of each mouse to form the primary tumor. Seven days later, the same cells were injected into the left inguinal region to induce a distant tumor. Mice were randomly allocated into seven groups (*n* = 5). The primary tumors were treated as described above, whereas no intervention was applied to the distant tumors. The volumes of bilateral tumors were recorded every 2 days between days 7 and 21. At 21 days after tumor cell inoculation, the remaining mice were sacrificed, and bilateral tumors were harvested and photographed for tumor size documentation.

**Anti-metastasis analysis in vivo**

To establish orthotopic liver tumor-bearing mouse models, 100 μL cell suspension was subcutaneously injected into the right flank of BALB/c and C57BL/6 mice, respectively. Specifically, BALB/c mice received suspension containing 5 × 10^6^ H22-Luc cells, while C57BL/6 mice were injected with an equal volume of Hepa1-6-Luc cell suspension. Once the tumor volume reached approximately 500 mm^3^, mice were sacrificed and the H22-Luc tumor tissues were excised. The tumor tissues were cut into small fragments of approximately 1 mm^3^ and surgically implanted into the livers of healthy BALB/c mice to generate an orthotopic hepatocellular carcinoma model. Lung metastasis was evaluated 42 days after treatment.

**Bioluminescent imaging and hematoxylin-eosin (HE) staining**

All mice were euthanized 15 minutes after intraperitoneal injection of fluorescein, and the lungs were removed for bioluminescence imaging.

Then the lung tissue was fixed with 4% paraformaldehyde at 4℃ overnight. The intact lungs were then embedded in paraffin and sectioned. Lung tissue sections were dewaxed and rehydrated, and then stained with hematoxylin-eosin (HE) following the instructions of the Hematoxylin and Eosin Staining Kit (Beyotime Biotechnology, Shanghai, China).

**Combined with PD-1 antibody therapy**

Hepa1-6 tumor cells (1 × 10^6^) were injected subcutaneously into the inguinal area of 6-week-old female C57BL/6 mice to establish a subcutaneous tumor model. These mice were divided at random into 4 groups (*n* = 5): control (200 μL, PBS), anti-PD-1 antibody (100 μg), BiO_2-X_/siFn1 + US (200 μL, BiO_2-X_ 5 mg kg^−1^, siFn1 133.3 μg kg^−1^, US 1.0 MHz, 1.0 W cm^−2^, 50% duty cycle, 3 min), BiO_2-X_/siFn1+US combined with anti-PD-1 antibody (200 μL, BiO_2-X_ 5 mg kg^−1^, siFn1 133.3 μg kg^−1^, US 1.0 MHz, 1.0 W cm^−2^, 50% duty cycle, 3 min, anti-PD-1 antibody 100 μg). The designated formulations were injected on days 7, 9, 11, and 13 following tumor inoculation, while US irradiation was applied to the corresponding treatment groups on days 8, 10, 12, and 14. Tumor volume and body weight were recorded every 2 days between days 5 and 30. After the end of treatment, the mice were euthanized (*n* = 5) and the tumors were dissected and weighed.

**Statistical analysis**

Statistical analyses were carried out using GraphPad Prism software (version 9.0.0; GraphPad Software, San Diego, CA, USA), and differences among multiple groups were evaluated by one-way analysis of variance (ANOVA), followed by Tukey’s post hoc test for multiple pairwise comparisons when the overall ANOVA showed statistical significance. Two-way ANOVA was used to evaluate the effects of two independent categorical variables on a continuous dependent variable; statistical comparisons were performed with Geisser–Greenhouse correction, and Tukey’s multiple comparisons test was applied as post hoc analysis to identify statistically significant differences between groups. And for other comparisons, unpaired Student’s t-test was used when comparing two groups. Data represent means ± standard deviation (SD) in each figure as indicated. The p-value less than 0.05 was considered significant. **P* < 0.05, ***P* < 0.01, ****P* < 0.001, and *****P* < 0.0001.

**Supporting Figures**

**Figure S1**. **(a)** RNAseq-based KEGG analysis of differential gene expression profiles after PD-1 antibody treatment resistance (*n* = 4). Statistical difference was calculated using Fisher’s exact test. **(b)** FN expression in shControl and shFn1 Hepa1-6 tumor cells by Western blot (n = 3). **(c)** Quantitative analysis of FN expression (*n* = 3). **(d)** Schematic illustration of the anti-PD-1 treatment regimen. **(e)** Tumor growth curves in the mouse model (*n* = 5). **(f)** 3D images of the current signal distribution collected during electrical AFM of BiO_2-X_ NSs. **(g)** EDS spectrum of BiO_2-X_. **(h)** EDS spectrum of BiO_2-X_/siFn1. **(i)** The sonothermal properties of H_2_O under different US power irradiation. **(j)** Corresponding thermal images of H_2_O after irradiation with US. Statistical significance for **c** was determined using a two-tailed unpaired Student’s t-test. Data were expressed as means ± SD. *p < 0.05, **p < 0.01, ***p < 0.001, ****p < 0.0001, n.s., no significance.

**Figure S2**. **(a)** The effects of BiO_2-X_/siFn1 on the expression of FN protein at different temperatures. **(b)** Quantitative analysis of FN expression (*n* = 3). **(c)** Agarose gel electrophoresis of BiO_2-X_/siFn1 after treatment at different temperatures. **(d)** The time-dependent release curves of siFn1 from BiO_2-X_/siFn1 at different temperatures (*n* = 3). **(e)** RNase stability of BiO_2-X_/siFn1 at different time. **(f)** The effects of BiO_2-X_/siFn1 on FN protein expression under different physiological buffer conditions. **(g)** Quantitative analysis of FN expression (*n* = 3). UV–vis absorption spectra for ROS detection with DPBF as the indicator: **(h)** DPBF + US group, **(i)** DPBF + BiO_2-X_ + US group, **(j)** DPBF + BiO_2-X_/siFn1 + US group. **(k)** Comparison of the oxidation of DPBF + US, DPBF + BiO_2-X_ + US, and DPBF + BiO_2-X_/siFn1 + US. For panel **d**, statistical differences were assessed by two-way ANOVA with Geisser–Greenhouse correction, followed by Tukey’s multiple comparisons test for post hoc analysis to determine statistical significance; statistical analyses for panels **b** and **g** were performed using one-way ANOVA, followed by Tukey’s post hoc test for multiple pairwise comparisons when the overall ANOVA showed statistical significance. Data were expressed as means ± SD. *p < 0.05, **p < 0.01, ***p < 0.001, ****p < 0.0001, n.s., no significance.

**Figure S3**. **(a)** Representative CLSM images of NIH-3T3 cells with DAPI and Cy5.5-labeled BiO_2-X_/siFn1. **(b)** Representative CLSM images of Hepa1-6 cells with DAPI and Cy5.5-labeled siFn1. **(c)** Bioluminescence imaging of Hepa1-6-Luc cells after 24 h of treatment with BiO_2-X_ NSs loaded with the indicated concentrations of siLuc. **(d)** The graph shows the average radiance of the cells (*n* = 3). **(e)** The expression of FN protein in different treatment groups. **(f)** Quantitative analysis of FN expression (*n* = 3). Statistical differences were calculated using a one-way ANOVA, followed by Tukey’s post hoc test for multiple pairwise comparisons when the overall ANOVA showed statistical significance. Data were expressed as means ± SD. *p < 0.05, **p < 0.01, ***p < 0.001, ****p < 0.0001, n.s., no significance.

**Figure S4**. **(a)** Fluorescence images of live/dead staining and **(b)** relative cell viabilities of Hepa1-6 cells after treatments (*n* = 3). Control (G1), US (G2), BiO_2-X_ (G3), siFn1 (G4), BiO_2-X_/siFn1 (G5), BiO_2-X_ + US (G6), and BiO_2-X_/siFn1 + US (G7). Data were expressed as means ± SD.

**Figure S5**. **(a)** Flow cytometry analysis of CRT expression in Hepa1-6 cells following various treatments. **(b)** Quantitative analysis of CRT expression (*n* = 3). **(c)** HMGB1 secretion measured by HMGB1 assay kits (*n* = 3). **(d)** Gating strategies for mature DCs. Statistical differences were calculated using a one-way ANOVA, followed by Tukey’s post hoc test for multiple pairwise comparisons when the overall ANOVA showed statistical significance. Control (G1), US (G2), BiO_2-X_ (G3), siFn1 (G4), BiO_2-X_/siFn1 (G5), BiO_2-X_ + US (G6), and BiO_2-X_/siFn1 + US (G7). Data were expressed as means ± SD. *p < 0.05, **p < 0.01, ***p < 0.001, ****p < 0.0001, n.s., no significance.

**Figure S6**. **(a)** Hemolytic pictures and **(b)** hemolytic rates of BiO_2-X_/siFn1 with different concentrations incubated with plasma for 1 h or 4 h, respectively (*n* = 3). **(c)** HE staining of the major organs harvested from Hepa1-6 tumor-bearing mice after treatment at different time points. **(d)** In vivo hematological indices. Hematological assays of mice at 30 days after BiO_2-X_/siFn1 injection (*n* = 4). Negative control (NC), a group without any treatment. Statistical differences were calculated using a one-way ANOVA, followed by Tukey’s post hoc test for multiple pairwise comparisons when the overall ANOVA showed statistical significance. Data were expressed as means ± SD. *p < 0.05, **p < 0.01, ***p < 0.001, ****p < 0.0001, n.s., no significance.

**Figure S7**. **(a)** In vivo liver and kidney function index. Hematological assays of mice 30 days after BiO_2-X_/siFn1 injection (*n* = 4). **(b)** Bi distribution and excretion profiles in harvested organs, **(c)** urine, and **(d)** feces post-treatment with PBS or BiO_2-X_/siFn1 were determined via ICP-MS elemental analysis (*n* = 3). Bi content is expressed as the mass of Bi per gram of harvested organ or body fluid from mice. For panel **b**, statistical significance was analyzed using two-way ANOVA with Tukey's multiple comparisons test. For panel **a**, **c**, and **d**, statistical differences were calculated using a one-way ANOVA, followed by Tukey’s post hoc test for multiple pairwise comparisons when the overall ANOVA showed statistical significance. Data were expressed as means ± SD. *p < 0.05, **p < 0.01, ***p < 0.001, ****p < 0.0001, n.s., no significance.

**Figure S8**. **(a)** Ex vivo fluorescence imaging of major organs, including the heart, liver, spleen, lung, kidney, and tumor in C57BL/6 mice after various treatments as indicated at 24 h. **(b)** Quantification of the fluorescence signal in the tumor after different treatment at 24 h (*n* = 3). **(c)** Schematic diagram of experimental plan. **(d)** Variation in tumor volume among mice in the various administration groups (*n* = 5). **(e)** Individual tumor growth curves of Hepa1-6-bearing mice after treatment (*n* = 5). **(f)** Individual tumor growth curves of Hepa1-6-bearing mice after treatment with different groups (*n* = 10). For panel **d**, statistical differences were assessed by two-way ANOVA with Geisser–Greenhouse correction, followed by Tukey’s multiple comparisons test for post hoc analysis to determine statistical significance; statistical analyses for panel **b** were performed using one-way ANOVA, followed by Tukey’s post hoc test for multiple pairwise comparisons when the overall ANOVA showed statistical significance. Control (G1), US (G2), BiO_2-X_ (G3), siFn1 (G4), BiO_2-X_/siFn1 (G5), BiO_2-X_ + US (G6), and BiO_2-X_/siFn1 + US (G7). Data were expressed as means ± SD. *p < 0.05, **p < 0.01, ***p < 0.001, ****p < 0.0001, n.s., no significance.

**Figure S9**. **(a)** Body weight of individual Hepa1-6 tumor-bearing mice (*n* = 10). **(b)** Average body weight of Hepa1-6 tumor-bearing mice in different treatment groups (*n* = 10). **(c)** Survival curves of Hepa1-6-tumor-bearing mice with different treatment (*n* = 10, Statistical differences of survival were calculated using Log-rank test). **(d)** AFM stiffness maps of tumors. **(e)** Histogram showing the distribution of measured Young’s modulus of tumor. Control (G1), US (G2), BiO_2-X_ (G3), siFn1 (G4), BiO_2-X_/siFn1 (G5), BiO_2-X_ + US (G6), and BiO_2-X_/siFn1 + US (G7). Data were expressed as means ± SD. *p < 0.05, **p < 0.01, ***p < 0.001, ****p < 0.0001, n.s., no significance.

**Figure S10**. **(a)** Different channels of immunofluorescence images of CD3^+^CD4^+^ and CD3^+^CD8^+^ **(b)** T cells of tumor tissue after different treatment. Control (G1), US (G2), BiO_2-X_ (G3), siFn1 (G4), BiO_2-X_/siFn1 (G5), BiO_2-X_ + US (G6), and BiO_2-X_/siFn1 + US (G7).

**Figure S11**. **(a)** IF imaging of CRT and HMGB1 staining in tumor tissues after various treatments. **(b)** Quantification of CRT Mean fluorescence intensity (MFI) (*n* = 3). **(c)** Quantification of nuclear HMGB1 MFI (*n* = 3). **(d)** Gating strategies for isolating CD80^+^CD86^+^ mature DCs from tumor tissue. **(e)** The quantitative analysis of mature DCs in tumor tissue after different treatments (*n* = 3). **(f)** Gating strategies for isolating CD4^+^ and CD8^+^ T cells from spleen tissue. **(g)** The quantification of CD4^+^ and CD8^+^ T cells in the spleen after different treatments (*n* = 3). **(h)** The quantification of CD3^+^CD4^+^ T cells in the spleen after different treatments (*n* = 3). **(i)** The quantification of CD3^+^CD8^+^ T cells in the spleen after different treatments (*n* = 3). Statistical differences were calculated using a one-way ANOVA, followed by Tukey’s post hoc test for multiple pairwise comparisons when the overall ANOVA showed statistical significance. Control (G1), US (G2), BiO_2-X_ (G3), siFn1 (G4), BiO_2-X_/siFn1 (G5), BiO_2-X_ + US (G6), and BiO_2-X_/siFn1 + US (G7). Data were expressed as means ± SD. *p < 0.05, **p < 0.01, ***p < 0.001, ****p < 0.0001, n.s., no significance.

**Figure S12**. **(a)** Gating strategies for isolating Tregs from tumor tissue. **(b)** The quantitative analysis of Tregs in primary tumor tissue after different treatments (*n* = 3). **(c)** Corresponding growth curves of primary tumors in different groups (*n* = 5). **(d)** The weight of primary tumors in different treatment groups (*n* = 5). Statistical differences were calculated using a one-way ANOVA, followed by Tukey’s post hoc test for multiple pairwise comparisons when the overall ANOVA showed statistical significance. Control (G1), US (G2), BiO_2-X_ (G3), siFn1 (G4), BiO_2-X_/siFn1 (G5), BiO_2-X_ + US (G6), and BiO_2-X_/siFn1 + US (G7). Data were expressed as means ± SD. *p < 0.05, **p < 0.01, ***p < 0.001, ****p < 0.0001, n.s., no significance.

**Figure S13**. **(a)** Gating strategies for isolating T cells (CD3^+^CD8^+^CD44^+^CD62L^−^) (Tem) and (CD3^+^CD8^+^CD44^+^CD62L^+^) (Tcm). **(b)** Diagrammatic representation of the in vivo experimental workflow using BALB/c mice for evaluating the anti-metastatic effect of BiO_2-X_/siFn1 + US. **(c)** Representative bioluminescence imaging and HE-stained images of lungs with metastatic lesions. **(d)** Bioluminescence signal intensity quantification of metastatic lung nodules in the H22 tumor model (*n* = 3). **(e)** Quantitative assessment of pulmonary metastatic nodules (*n* = 5). **(f)** Lung weight of excised lungs in each group (*n* = 5). **(g)** Typical flow cytometry profiles of splenic effector memory T cells (Tem) and central memory T cells (Tcm) after various treatments. **(h)** Quantification of Tem and Tcm in the spleen after various treatments (*n* = 3). Statistical differences were calculated using a one-way ANOVA, followed by Tukey’s post hoc test for multiple pairwise comparisons when the overall ANOVA showed statistical significance. Control (G1), US (G2), BiO_2-X_ (G3), siFn1 (G4), BiO_2-X_/siFn1 (G5), BiO_2-X_ + US (G6), and BiO_2-X_/siFn1 + US (G7). Data were expressed as means ± SD. *p < 0.05, **p < 0.01, ***p < 0.001, ****p < 0.0001, n.s., no significance.
